# Dynamics of recombination *via* conical intersection in a semiconductor nanocrystal†
†Electronic supplementary information (ESI) available. See DOI: 10.1039/c7sc04221c


**DOI:** 10.1039/c7sc04221c

**Published:** 2017-11-13

**Authors:** Wei-Tao Peng, B. Scott Fales, Yinan Shu, Benjamin G. Levine

**Affiliations:** a Department of Chemistry , Michigan State University , East Lansing , MI 48824 , USA . Email: levine@chemistry.msu.edu; b Department of Chemistry , The PULSE Institute , Stanford University , Stanford , CA 94305 , USA; c SLAC National Accelerator Laboratory , Menlo Park , CA 94025 , USA; d Department of Chemistry , University of Minnesota , Minneapolis , MN 55455 , USA

## Abstract

Conical intersections are well known to introduce nonradiative decay pathways in molecules, but have only recently been implicated in nonradiative recombination processes in materials. Here we apply excited state *ab initio* molecular dynamics simulations based on a multireference description of the electronic structure to defective silicon nanocrystals up to 1.7 nm in diameter to search for accessible nonradiative recombination pathways. Dangling bond defects are found to induce conical intersections between the ground and first excited electronic states of five systems of various sizes. These defect-induced conical intersections are accessible at energies that are in the visible range (2.4–2.7 eV) and very weakly dependent on particle size. The dynamic simulations suggest that these intersections are accessed 40–60 fs after creation of a defect-localized excitation. This ultrafast recombination is attributed to the fact that Jahn–Teller distortion on the first excited state drives the defect directly towards a conical intersection with the ground electronic state.

## Abstract 2

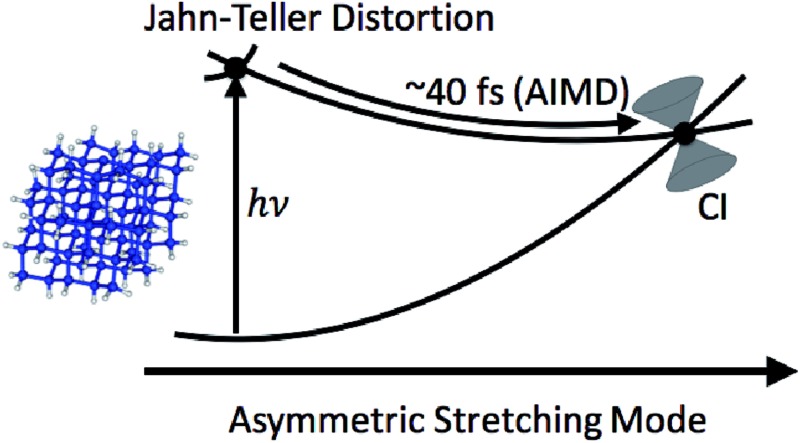
The ultrafast dynamics of nonradiative recombination at dangling bond defects is elucidated by nanoscale multireference *ab initio* molecular dynamics simulations.

## Introduction

Conical intersections (CIs) are points of degeneracy between the potential energy surfaces (PESs) of two or more adiabatic electronic states.^1^–^6^ It is now well established that molecules that undergo efficient, ultrafast nonradiative transitions between electronic states of the same spin often do so by passing through CIs connecting those states. CIs can therefore be thought of in analogy to transition states; transition states are representative of paths that connect reactant and product wells on the same PES, whereas CIs are representative of nonradiative pathways connecting different electronic states. Theoretical predictions of CIs have provided insights into many important photochemical phenomena, *e.g.* photoisomerization,^4^,^7^ photodissociation,^8^–^10^ vision,^11^,^12^ and nonradiative decay of nucleic acids.^13^,^14^ Identification of CIs is now a routine part of the computational molecular photochemistry toolbox.^15^ However, only recently and through the use of advanced computing technology^16^ has the role that CIs play in the nonradiative recombination of excitations in semiconductors come to light.^17^,^18^

Here we investigate the role of CIs in the photophysics of silicon nanocrystals (SiNCs) with dangling bond defects. SiNCs and other low-dimensional silicon systems have received intense attention due to their unique photophysical properties. Unlike bulk silicon, which has an indirect band gap, low-dimensional silicon materials can efficiently emit visible light^19^ with a wavelength that can be tuned *via* quantum confinement^20^–^23^ or surface modification.^24^–^27^ This tunable emission enables their application in optoelectronic devices,^28^–^30^ biological imaging,^31^ and silicon lasers.^32^,^33^ Under ambient conditions, however, SiNCs are prone to oxidize quickly upon exposure to O_2_ and/or H_2_O. Oxidation generates various defects on the surfaces of SiNCs, including dangling bonds (DBs) and some silicon oxide species (Si–O–Si bridges and Si–OH). Among them, silicon dangling bond defects have been studied extensively.^34^–^38^ In general, there are two common types of dangling bond defects: P_b_ centers, which are dangling bonds on a three-coordinated silicon atom on the surface, and D centers, which are DB defects located in amorphous silicon.^39^ DB defects have been known to degrade the performance of silicon-based devices for both photovoltaic^40^,^41^ and light emission^38^,^42^–^44^ applications.

Thus, it is well-known that silicon DB defects are nonradiative centers in SiNCs. It is also established that electronic movement at DB centers is strongly coupled to local vibrational motions.^45^,^46^ The widely accepted mechanism for recombination involves the sequential capture of electron and hole into the non-bonding orbital of the P_b_ center. Each change in the oxidation state of the P_b_ center is accompanied by nuclear relaxation along a bending mode that maintains local *C*_3v_ symmetry; reduction results in the defect silicon atom taking on a less planar structure (*i.e.* less sp^2^-like) while oxidation results in a more planar structure. The line of thinking that yields this mechanism arises from the assumption that each change in charge state is instantaneous, however. It neglects (a) the fact that electrons and holes are strongly confined in SiNCs and therefore may interact strongly with one another and with the defect site before localization to the P_b_ center, and (b) the interaction of electron and hole with the defect is not instantaneous and may, instead, involve complex electron-nuclear dynamics. The CI theory of recombination considers these interactions and dynamics explicitly, therefore it would be instructive to reinvestigate this recombination process from a CI point of view.

In this work we (a) investigate whether nonradiative dynamics at P_b_ centers can be attributed to CIs between the ground and first excited electronic states, and (b) inform our fundamental physical intuition for recombination processes in general by analysis of the CIs associated with the dangling bond defect. To these ends we will bring to bear novel *ab initio* molecular dynamics (AIMD) tools capable of modeling the dynamics of electronically excited SiNCs as they approach CIs with the ground electronic state. In AIMD simulations, the nuclear dynamics are computed on PESs that are solved on the fly *via* electronic structure calculations. AIMD has recently become a tool of choice for the theoretical study of the photophysics of nanomaterials when either direct knowledge of excited state dynamics or extensive thermodynamic sampling are required, shedding light on various aspects of the charge carrier dynamics of SiNCs.^47^–^56^ The current study is the first to apply an AIMD approach based on a multireference description of the electronic structure to a true nanocrystal (diameter 1.7 nm). The advantage of multireference electronic structure approaches such as the complete active space configuration interaction (CASCI) approach used here^57^,^58^ is that they can accurately describe the PES in the vicinity of CIs between the ground and first excited electronic states. This is in contrast to single reference electronic structure methods—such as time-dependent density functional theory—which cannot accurately describe the potential energy surface near CIs involving the ground electronic state.^59^ The below study illustrates how applying CASCI-AIMD to model the dynamics of a semiconductor nanocluster from excitation to the neighborhood of a conical intersection can inform our fundamental understanding of nonradiative recombination.

## Results and discussion

**Fig. 1:**
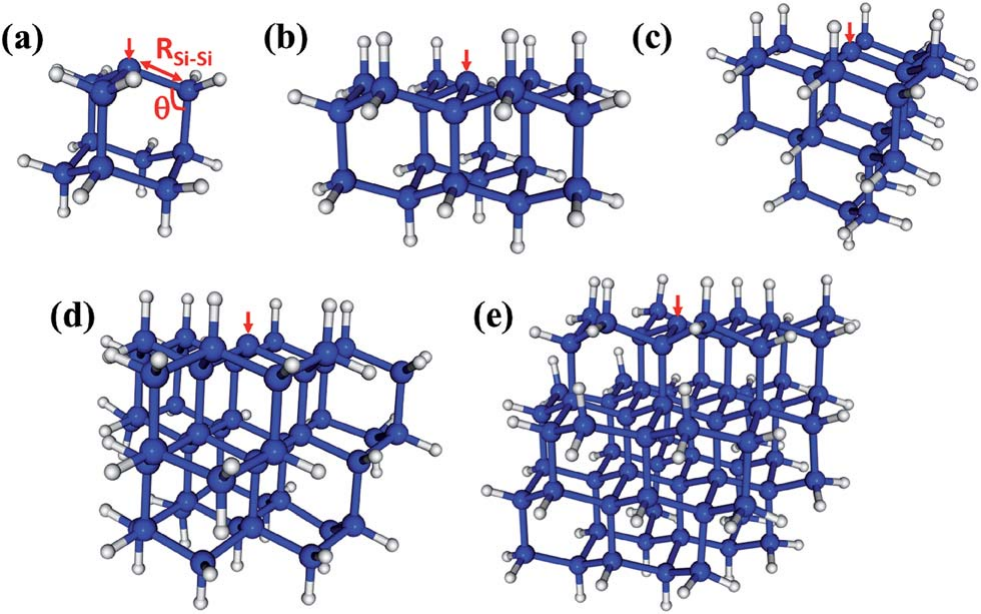
The P_b_-containing silicon clusters studied in this work. (a) Si_10_H_15_ (sila-adamantane cluster), (b) Si_22_H_27_, (c) Si_26_H_31_, (d) Si_47_H_49_ (1.3 nm SiNC), and (e) Si_72_H_63_ (1.7 nm SiNC). One of the three symmetry-equivalent Si–Si bond lengths (*R*_Si–Si_) and bond angles (*θ*) discussed herein are indicated in red in (a). The red arrows indicate the positions of dangling bond defects.

The methodological and computational specifics of our CASCI-AIMD simulations are presented in ESI,† but here we outline our study. We have performed a single CASCI-AIMD simulation for each of a series of five silicon clusters. Each cluster has a single P_b_ defect on the surface. The clusters (pictured in Fig. 1) range in size from a single sila-adamatane unit (Si_10_H_15_) to a 1.7 nm particle (Si_72_H_63_). All simulations were run on the first excited electronic PES starting from a structure in the Franck–Condon region. (Structures are presented in ESI.†) All surface silicon atoms aside from the defect site were capped with hydrogen atoms. We emphasize that these simulations are performed in the Born–Oppenheimer approximation.

Geometries of near-zero energy gap were drawn from the AIMD trajectories and the minimal energy CIs (MECIs; the local minima on the CI seam) were optimized. Because CASCI lacks dynamic electron correlation, complete active space second-order perturbation theory (CASPT2) calculations^60^ were performed on the smallest cluster to estimate errors in our CASCI energies. Static CASCI and CASCI-AIMD calculations were performed in the TeraChem software package,^16^,^61^–^63^ which enables these demanding calculations through the use of graphics processing units—high performance computer processors designed for graphical applications such as video games. CASPT2 calculations were performed in MolPro,^64^–^68^ coupled cluster calculations were performed in GAMESS,^69^–^71^ and conical intersection optimizations were performed with CIOpt.^72^ Through this work we prefer adiabatic state labels: D_0_ and D_1_ to indicate the ground and first excited spin doublet electronic states of the clusters, respectively. However, when useful and appropriate we also include term symbols ^2^A_1_ and ^2^E to reflect the approximate symmetry of the states with respect to the local *C*_3v_ symmetry of the defect site.

**Fig. 2:**
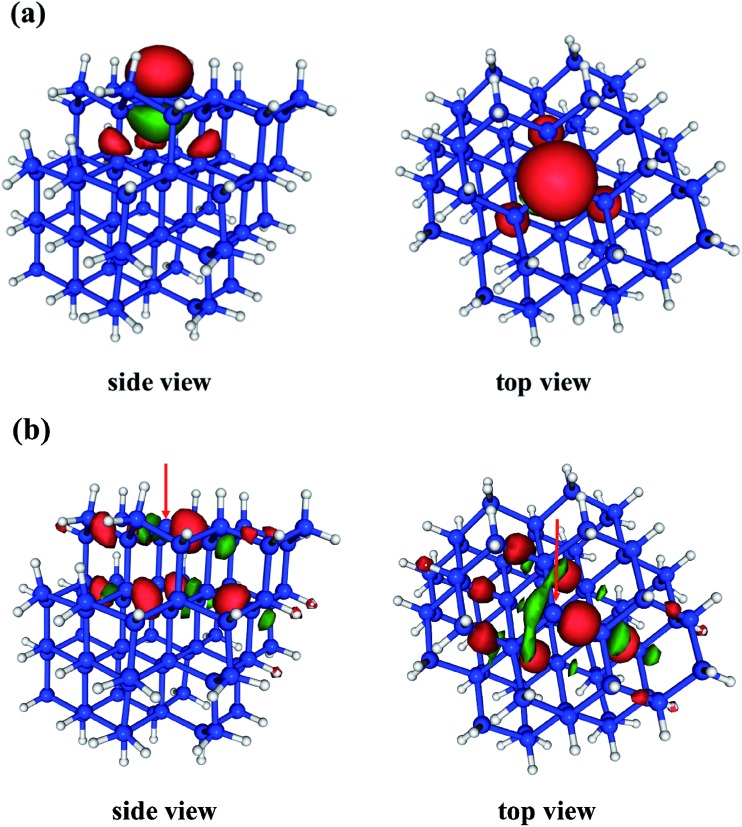
Orbitals representative of the D_0_ → D_1_ (^2^A_1_ → ^2^E) transition of the Si_72_H_63_ system. (a) The singly occupied molecular orbital (SOMO) is the nonbonding (n) orbital of the P_b_ center, and (b) the highest doubly occupied orbital (HDOMO) has Si–Si σ bonding character in the vicinity of the P_b_ defect (σ_Si–Si_). Note that this HDOMO is approximately degenerate due to the local symmetry of the defect. We show only one of the two nearly degenerate orbitals. The red arrows indicate the locations of the P_b_ center.

The D_0_ (^2^A_1_) minimum energy structures of our five clusters are presented in Fig. 1. Bond lengths and bond angles around the P_b_ defect (as indicated in Fig. 1a) at these structures are listed in the Table 1, labeled FC (Franck–Condon point). In these initial structures the three Si–Si bond lengths surrounding the DB and the associated bond angles are symmetric. The vertical excitation energies (Table 1) of the five clusters are very similar to one another, varying by only 0.3 eV, and there is not a strong trend with system size. This absence of quantum confinement effects suggests the locality of the excitation. This locality can be seen in the orbitals involved in the excitation (pictured in Fig. 2). The excitation occurs from the highest doubly occupied molecular orbital (HDOMO) to the singly occupied molecular orbital (SOMO). The HDOMO is a Si–Si σ bonding orbital (σ_Si–Si_; e), which is relatively local to the region of the defect, while the SOMO is the dangling bond itself: the nonbonding (n; a_1_) sp^3^ orbital of the defect silicon atom. Note that there is a nearly degenerate pair of HDOMOs due to the local *C*_3v_ symmetry of the defect. We present only one of the two degenerate σ_Si–Si_ orbitals in Fig. 2.

**Table 1:** The Si–Si bond lengths (Å), Si–Si–Si bond angles (*θ* as indicated in Fig. 1a; in degrees), and D_1_ energies of differently sized silicon clusters at the Franck–Condon point (FC) and MECI. All energies are relative to the D_0_ minimum energy. Energies are computed at the CASCI level of theory as described in ESI. When available, CASPT2 energies at CASCI geometries are presented in parenthesis

	FC			MECI		
	*R*_Si–Si_/Å	*θ*/degree	D_1_ energy/eV	*R*_Si–Si_/Å	*θ*/degree	D_1_ energy/eV
Si_10_H_15_	2.34	107.2	4.23 (3.53)	2.43	123.8	2.67 (2.69)
	2.34	107.2		2.45	123.7	
	2.34	107.2		2.94	124.0	
Si_22_H_27_	2.36	105.2	4.25	2.46	122.6	2.52
	2.36	105.2		2.46	121.9	
	2.36	105.2		2.89	123.5	
Si_26_H_31_	2.36	104.9	4.29	2.43	122.0	2.52
	2.36	104.9		2.51	123.2	
	2.36	104.9		2.83	123.6	
Si_47_H_49_	2.36	105.6	3.99	2.43	122.4	2.44
	2.36	105.6		2.54	123.6	
	2.36	105.6		2.81	123.6	
Si_72_H_63_	2.35	105.7	4.00	2.44	122.4	2.38
	2.35	105.7		2.48	122.9	
	2.35	105.7		2.82	124.3	

**Fig. 3:**
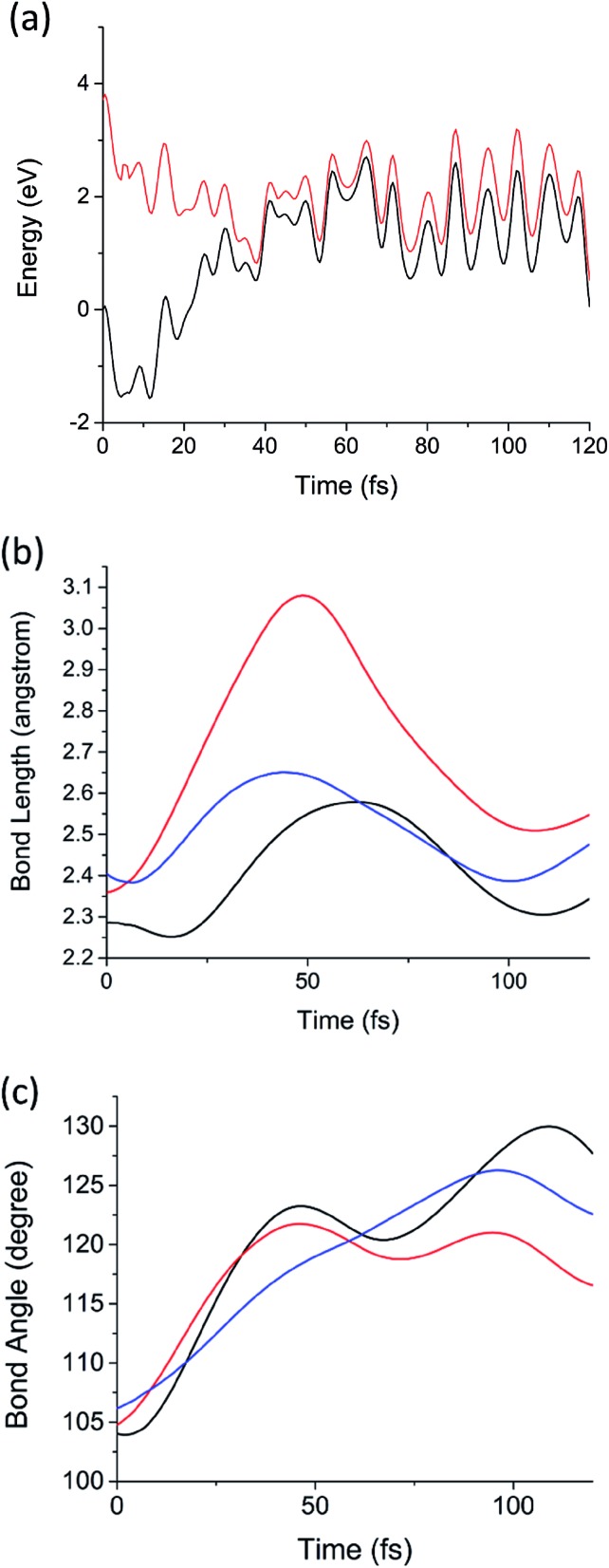
(a) The potential energies of the D_1_ (red) and D_0_ (black) electronic states as a function of time from the D_1_ AIMD simulation of Si_72_H_63_. (b) The three Si–Si bond lengths (*R*_Si–Si_) adjacent to the P_b_ defect as a function of time from the same AIMD calculation. (c) The three Si–Si–Si angles (*θ*, illustrated in Fig. 1a) as a function of time from the same AIMD calculation. Each color represents one of the three symmetry equivalent bond lengths or angles in (b) and (c), respectively.

Now we consider the results of the excited state AIMD simulations initiated on the D_1_ (^2^E; σ_Si–Si_ → n) state. This is the lowest electronic state with nonzero transition dipole moment. Note that D_1_ and D_2_ are degenerate by symmetry, as will be discussed below, and D_3_ is considerably higher in energy (0.9 eV above D_2_ in the sila-adamantane cluster). The time-dependent D_1_ and D_0_ potential energies, Si–Si bond lengths (*R*_Si–Si_ as defined in Fig. 1a), and bond angles (*θ* as illustrated in Fig. 1a) of the 1.7 nm SiNC are presented in Fig. 3. The dynamics of the smaller clusters were nearly identical; similar graphs for these cases are reported in Fig. S1–S12.† In all five cases the D_1_/D_0_ energy gap approaches zero (<0.1 eV) in the first 40–60 fs after excitation (Fig. 3a). The vanishing energy gaps strongly suggest the existence of low-lying D_1_/D_0_ CIs.^73^

Using low-gap structures from the AIMD trajectories as starting guesses, MECIs were optimized in all five systems. The energies and structural details of these MECIs are reported in Table 1. Full structures are presented in ESI.† Comparing the structures of MECIs to the Franck–Condon points, one can see that both bond lengths and bond angles around the DB defects increase at the MECIs. The three Si–Si bonds surrounding the DB are asymmetrically stretched in all five clusters; one Si–Si bond grows longer (2.81–2.94 angstrom) than the other two (2.43–2.54 angstrom). Similar asymmetric stretching is observed in the AIMD simulations of all five systems (Fig. 3b and S5–S8†) on the same 40–60 fs time scale on which the D_1_/D_0_ energy gap approaches zero. Much smaller changes in bond length are observed for Si–Si bonds not immediately adjacent to the P_b_ defect. Consistent with past work on dangling bond defects,^45^ symmetric bending motion is also observed to be important; the *θ* angles are observed to increase significantly both in the AIMD simulations (Fig. 3c and S9–S12†) and in the optimized MECI structures (Table 1). Taken together, these calculations suggest that upon excitation of the lowest defect-localized excited state, the P_b_ defect moves ballistically to the CI region in 40–60 fs. It is also noteworthy that the trajectories remain in a region of small energy gap after 40–60 fs, suggesting that it may pass over the intersection multiple times, enabling efficient decay.

**Fig. 4:**
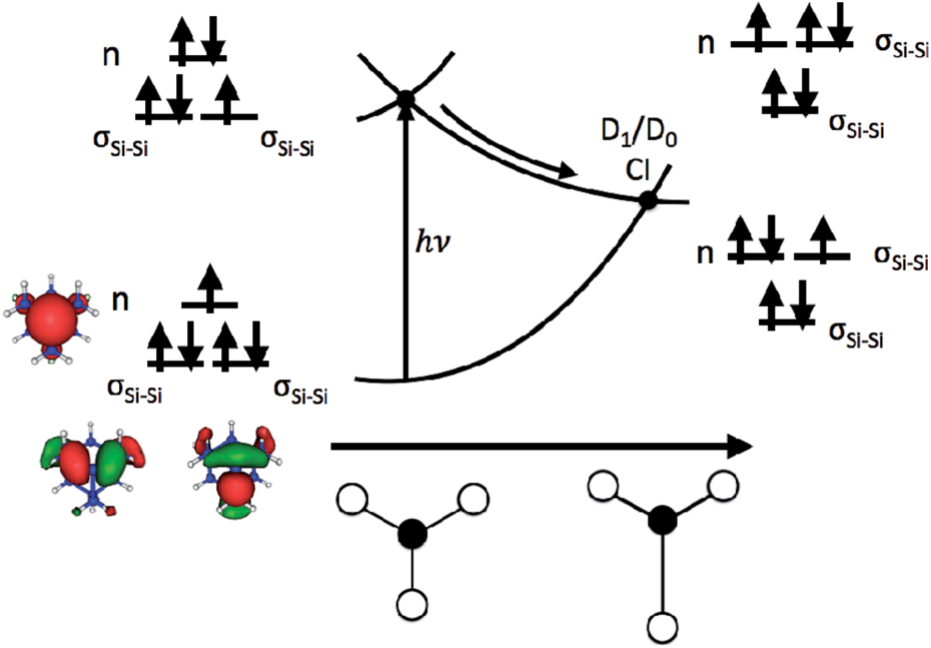
Schematic illustration of the dynamics of nonradiative recombination of an excitation at a P_b_ center. The PESs are plotted as a function of an asymmetric stretching coordinate about the P_b_ center (illustrated along the *x*-axis with the dangling bond site represented by a filled circle and the three adjacent silicon atoms represented by open circles). Insets show the orbital occupations of D_0_ and D_1_ and relative orbital energies at the FC point (left) and MECI (right). The n and σ_Si–Si_ orbitals of the smallest (sila-adamantane) system are shown on the bottom left.

The role that symmetry breaking plays in these nonradiative dynamics can be intuitively understood through analysis of the orbitals occupied during excitation. Fig. 4 is a schematic diagram summarizing these dynamics. As described above, excitation to D_1_ involves the promotion of an electron from one of the degenerate σ_Si–Si_ orbitals to the n orbital. This reduces the Si–Si bond order, resulting in a lengthening of one of the Si–Si bonds (as observed in the AIMD simulations of all five clusters). In the locally *C*_3v_-symmetric FC structure the D_1_ (^2^E) state is doubly degenerate by symmetry, thus this symmetry-breaking motion is a Jahn–Teller distortion. The lengthening of a single Si–Si bond (moving from left to right along the D_1_ PES in Fig. 4) brings the molecule towards the MECI structure. This symmetry breaking raises the energy of one of the σ_Si–Si_ orbitals into near degeneracy with the n orbital, bringing about a CI between the D_0_ and D_1_ states. That a Jahn–Teller distortion in D_1_ drives the molecules directly towards the D_1_/D_0_ CI provides a straightforward explanation for the ultrafast nonradiative process that follows creation of the defect-localized excitation.

The MECIs of the defective silicon clusters studied here are all accessible at energies in the visible range (2.38–2.67 eV above the ground state minimum structure; Table 1) and therefore capable of quenching visible light emission. The MECIs show a slight decrease in energy with increasing system size; the energy decreases from 2.67 eV for the small sila-adamantane cluster to 2.38 eV for the 1.7 nm SiNC. This small energy decrease of 0.29 eV is consistent with the localized nature of the defect-localized excited state. Calculations at the dynamically correlated CASPT2 level confirm the accuracy of the MECI energies predicted by CASCI, though vertical excitation energies are somewhat overestimated. All five clusters have D_1_ minimum energy structures distinct from the MECIs. In all cases this minimum is 0.05–0.06 eV below the MECI, compared to the 1.6–1.9 eV released during relaxation on the excited states. Thus the MECI is energetically accessible upon excitation. Energies and structures of D_1_ minima are presented in ESI.†

The existence of defect-induced CIs with energies in the 2.4–2.7 eV range is consistent with several experimental observations of the photoluminescence (PL) of SiNCs after oxidation. The MECI energies suggest that the PL of SiNCs with emission energies larger than ∼2.4–2.7 eV is likely to be quenched by the DB defects. Indeed the quantum yield of PL from oxidized SiNCs is observed to drop with increasing energy,^74^ and single particle experiments on oxidized SiNCs show no emissive particles with PL maxima above 2.5 eV.^75^ In addition, the PL lifetime of oxidized SiNCs decreases with increasing PL energy, and a dramatic decrease is observed in the 2.0–2.2 eV energy range.^76^ This decreasing PL lifetime suggests the existence of an efficient nonradiative recombination pathway accessible above these energies, consistent with our computed CI energies. Finally, as noted in our previous studies of oxygen-containing defects, the unusual size-insensitive orange (S-band) emission of oxidized SiNCs observed in ensemble PL measurements^77^ is consistent with the presence of CIs accessible in this energy range. We argue that the size-insensitivity of the observed ensemble emission arises not because the emission energy of individual oxidized SiNCs becomes insensitive to particle size, but instead because the rate of nonradiative recombination is strongly size sensitive, dramatically reducing the PL yields of smaller SiNCs with shorter wavelengths. This argument reconciles the observation of size-insensitive emission with PL lifetime, linewidth, and polarization measurements suggesting that the S-band arises from quantum-confined excitons.^75^,^78^,^79^

## Conclusions

Thus, we have elucidated the mechanism of nonradiative recombination *via* a dangling bond defect by application of AIMD simulations based on a multireference description of the electronic structure to SiNCs up to 1.7 nm in diameter. Within 40–60 fs after excitation of a defect-localized electronic excited state, a CI between the D_0_ and D_1_ states is accessed. This CI is accessible at energies in the 2.4–2.7 eV range, and thus is detrimental to visible PL, consistent with the fact that DBs are well-known nonradiative centers. The ultrafast recombination process is driven both by Jahn–Teller distortion in the D_1_ state and by totally symmetric bending of the DB center. The role that symmetry-breaking plays in this mechanism underlines the importance of treating coupled electron-nuclear dynamics in the study of recombination.

## Conflicts of interest

There are no conflicts to declare.

## Supplementary Material

Supplementary information
