# Supplementary material for: Dynamics of recombination via conical intersection in a semiconductor nanocrystal
Source: Chem Sci. 2017 Nov 13;9(3):681–7. doi: 10.1039/c7sc04221c (PMC5869574; doi:10.1039/c7sc04221c)
Supplement: Supplementary file 1 [file SC-009-C7SC04221C-s001.pdf]

Electronic Supplementary Material (ESI) for Chemical Science.
This journal is © The Royal Society of Chemistry 2017

Supplementary Information for "Dynamics of Recombination via Conical Intersection in a Semiconductor Nanocrystal"

Wei-Tao Peng[1], B. Scott Fales[2,3], Yinan Shu[4], Benjamin G. Levine[1*]
[1] Department of Chemistry, Michigan State University, East Lansing, MI 48824
[2] Department of Chemistry and the PULSE Institute, Stanford University, Stanford, CA 94305
[3] SLAC National Accelerator Laboratory, Menlo Park, CA 94025
[4] Department of Chemistry, University of Minnesota, Minneapolis, MN 55455

* to whom correspondence should be addressed: levine@chemistry.msu.edu

Methods

The floating occupations molecular orbitals complete active space configuration interaction (FOMO-CASCI) method provided the PESs for our AIMD simulations and for the ground state and MECI optimizations.[1-3] Three orbitals with five electrons are included in the active space. This active space is chosen by comparing excitation energies and excitation characters to the results of a higher level theory with no active space requirements: IP-EOM-CCSD/6-31G(d,p), for a sila-adamantane $Si_{10}H_{15}$ unit. The same active space is used for the larger clusters, assuming a similar excitation character. The LANL2DZ basis set and effective core potentials[4] (ECPs) were used for all FOMO-CASCI calculations. The initial positions and momenta for the AIMD calculations are sampled from the ground state vibrational Wigner distribution computed in the harmonic approximation. Sampled initial conditions were used rather than the Franck-Condon point to eliminate any artifacts associated with the high local symmetry of the Franck-Condon geometry. The classical equations of motion were integrated via the velocity Verlet algorithm with a time step of 0.5 fs. Complete active space second order perturbation theory calculations were performed at both the FC and MECI geometries of the smallest cluster to assess errors due to the absence of dynamics electronic correlation in the FOMO-CASCI calculations. These calculations utilized the LANL2DZdp polarized basis set and ECPs[4] and the same active space as above. Static multireference complete active space

configuration interaction calculations and AIMD were performed in the TeraChem software package,[3, 5-8] complete active space second order perturbation theory calculation were performed in MolPro,[9-11] coupled cluster calculations performed in GAMESS,[12-14] and conical intersection optimizations were performed with CIOpt.[15]

Supplementary Tables

Table S1. The FOMO-CASCI D_1 energies of the Franck-Condon (FC), D_1/D_0 MECI, and D_1 minimum geometries. All energies are relative to the ground states (D_0) minimum energy.

Cluster	FC D_1 En. (eV)	MECI D_1 En. (eV)	D_1 Min. D_1 En. (eV)
$Si_{10}H_{15}$	4.23	2.67	2.61
$Si_{22}H_{27}$	4.25	2.52	2.46
$Si_{26}H_{31}$	4.29	2.52	2.46
$Si_{47}H_{49}$	3.99	2.44	2.38
$Si_{72}H_{63}$	4.00	2.38	2.33

Supplementary Figures

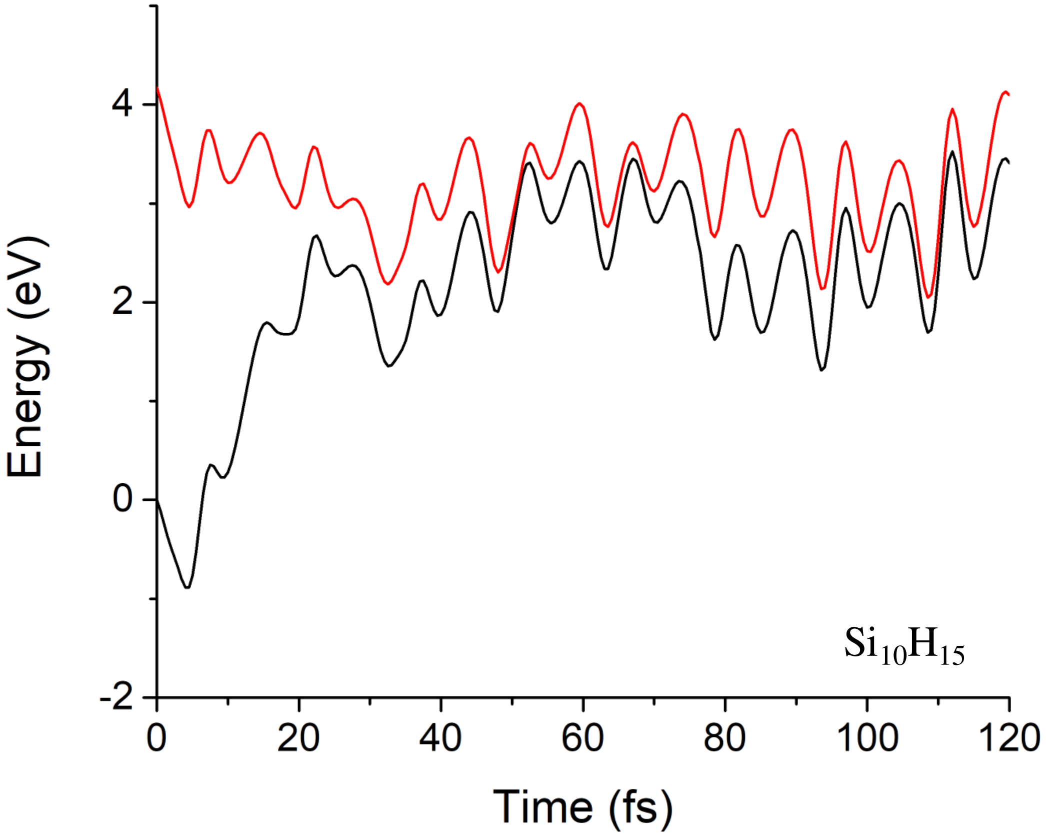

Figure S1. The potential energies of the first excited (red line) and ground (black line) states as a function of time from the AIMD simulation of $Si_{10}H_{15}$.

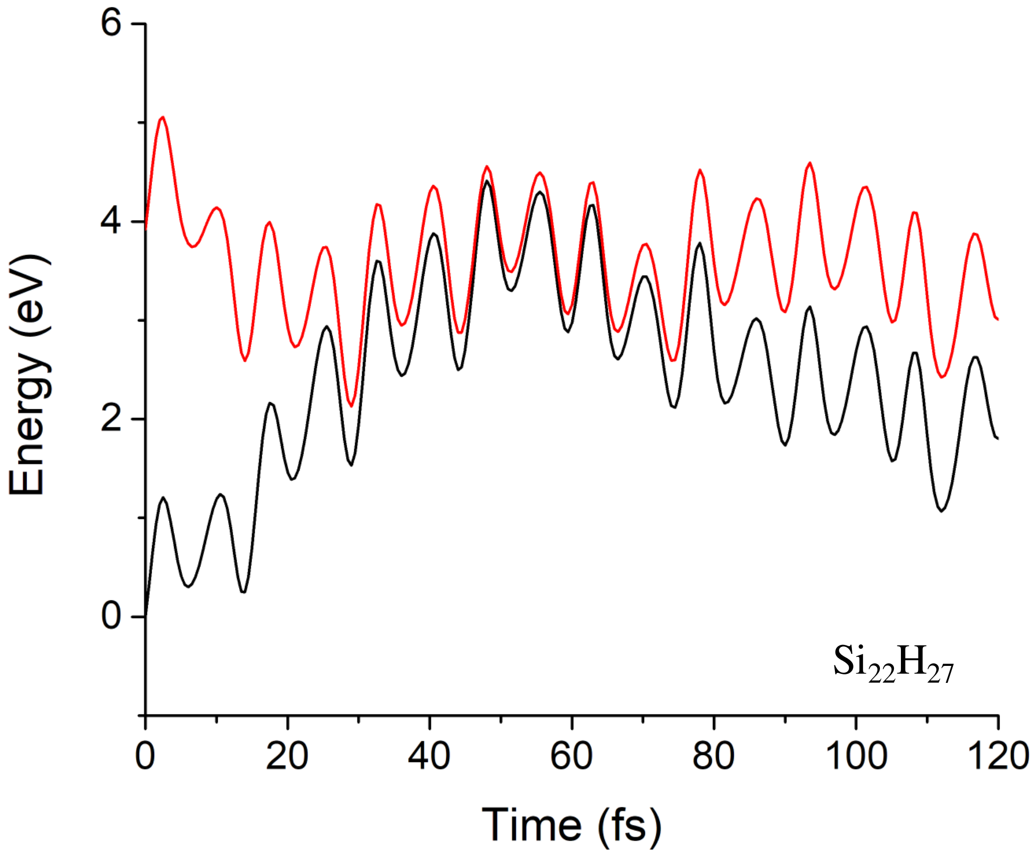

Figure S2. The potential energies of the first excited (red line) and ground (black line) states as a function of time from the AIMD simulation of $Si_{22}H_{27}$.

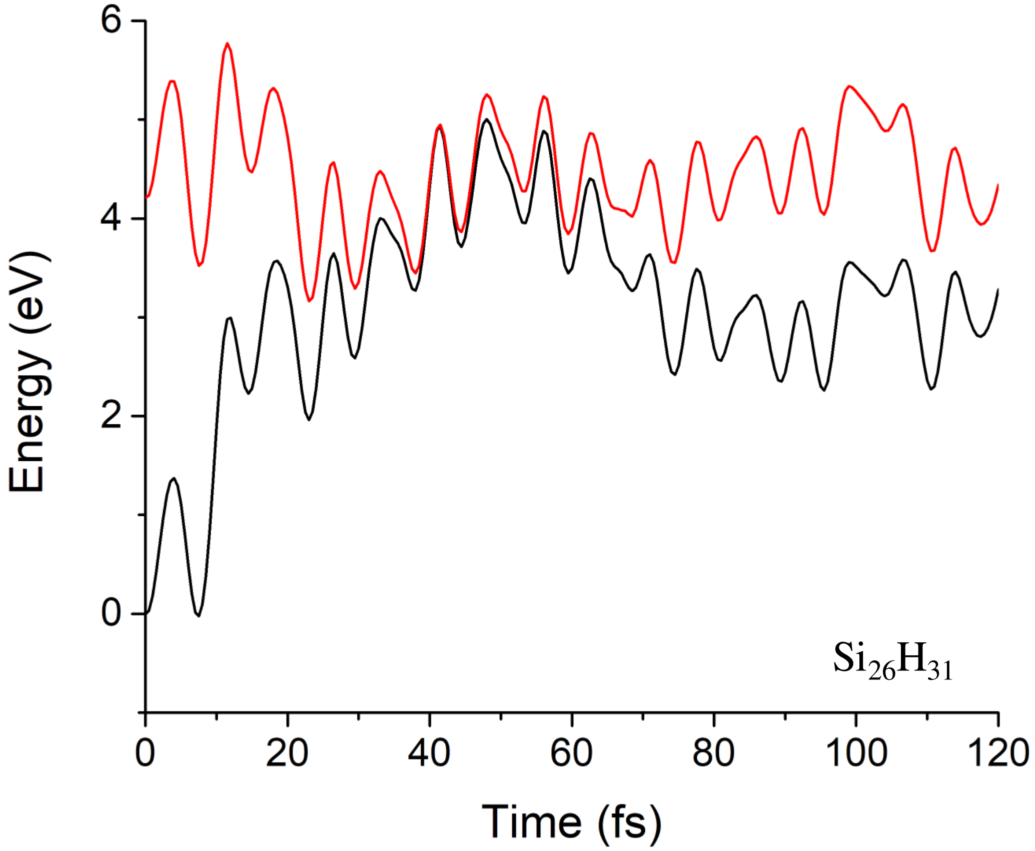

Figure S3. The potential energies of the first excited (red line) and ground (black line) states as a function of time from the AIMD simulation of $Si_{26}H_{31}$.

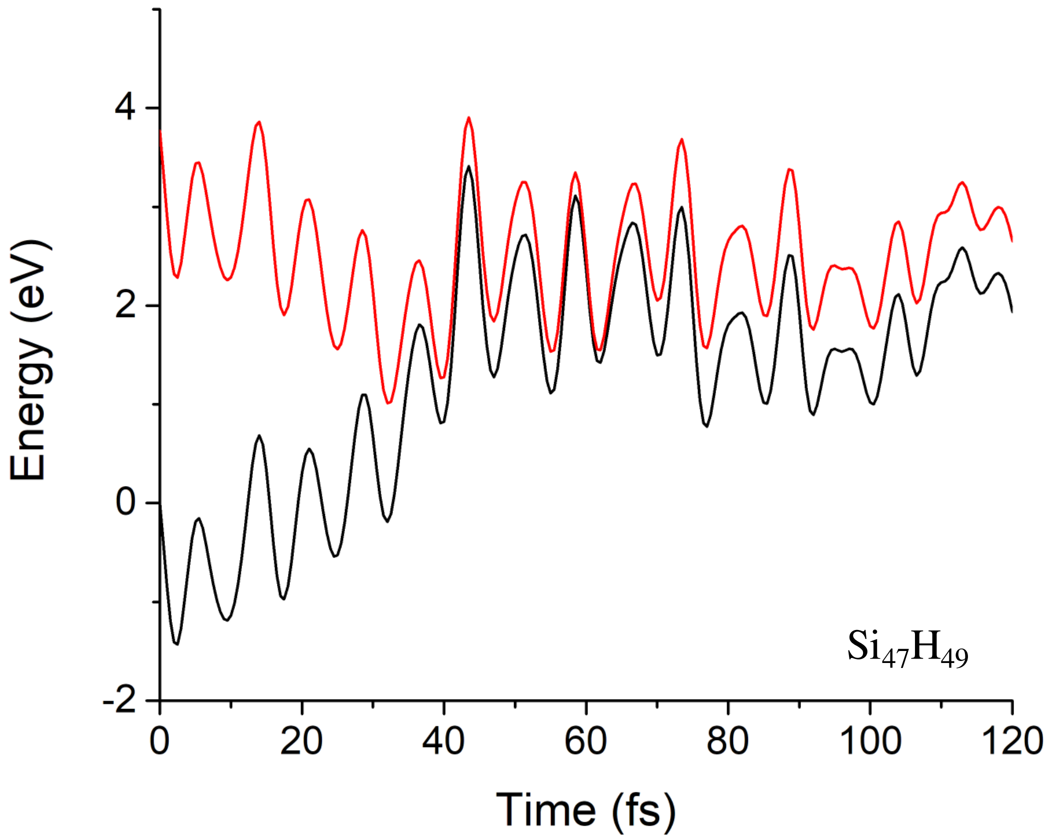

Figure S4. The potential energies of the first excited (red line) and ground (black line) states as a function of time from the AIMD simulation of $Si_{47}H_{49}$.

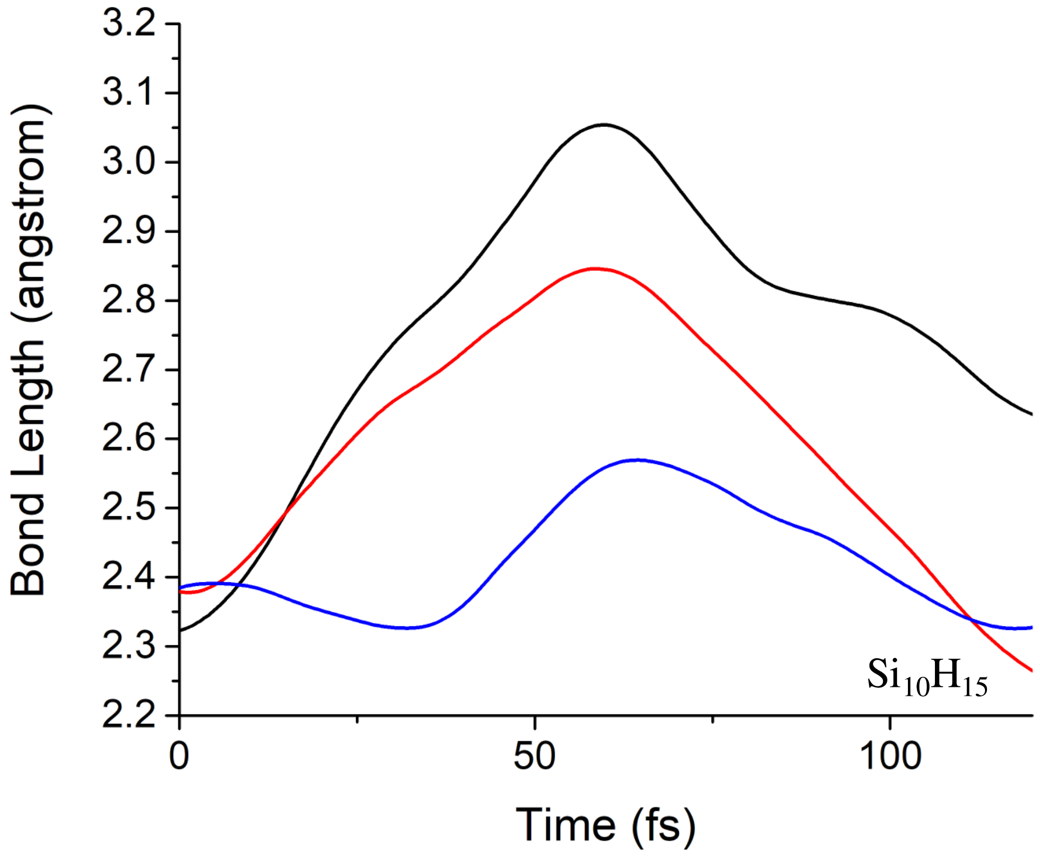

Figure S5. The three Si-Si bond lengths ($R_{Si\text{-}Si}$) as a function of time from the AIMD simulation of $Si_{10}H_{15}$. Each color represents one Si-Si bond length.

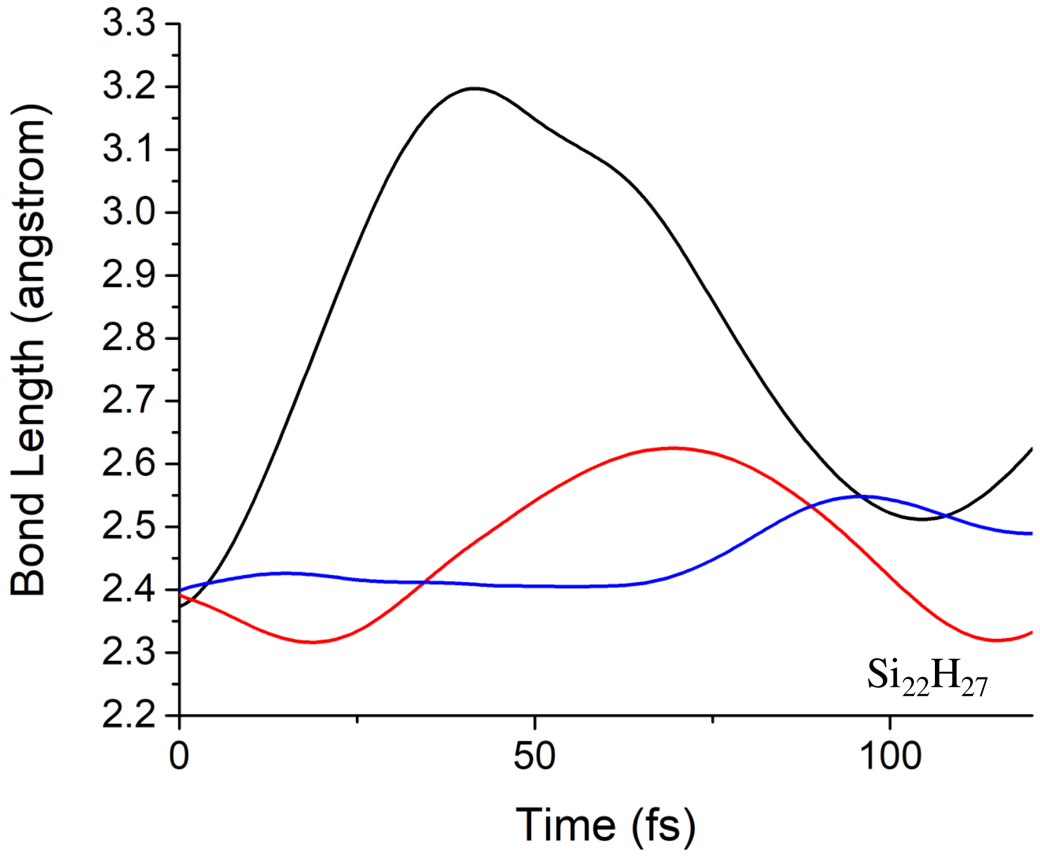

Figure S6. The three Si-Si bond lengths (R_{Si-Si}) as a function of time from the AIMD simulation of $Si_{22}H_{27}$. Each color represents one Si-Si bond length.

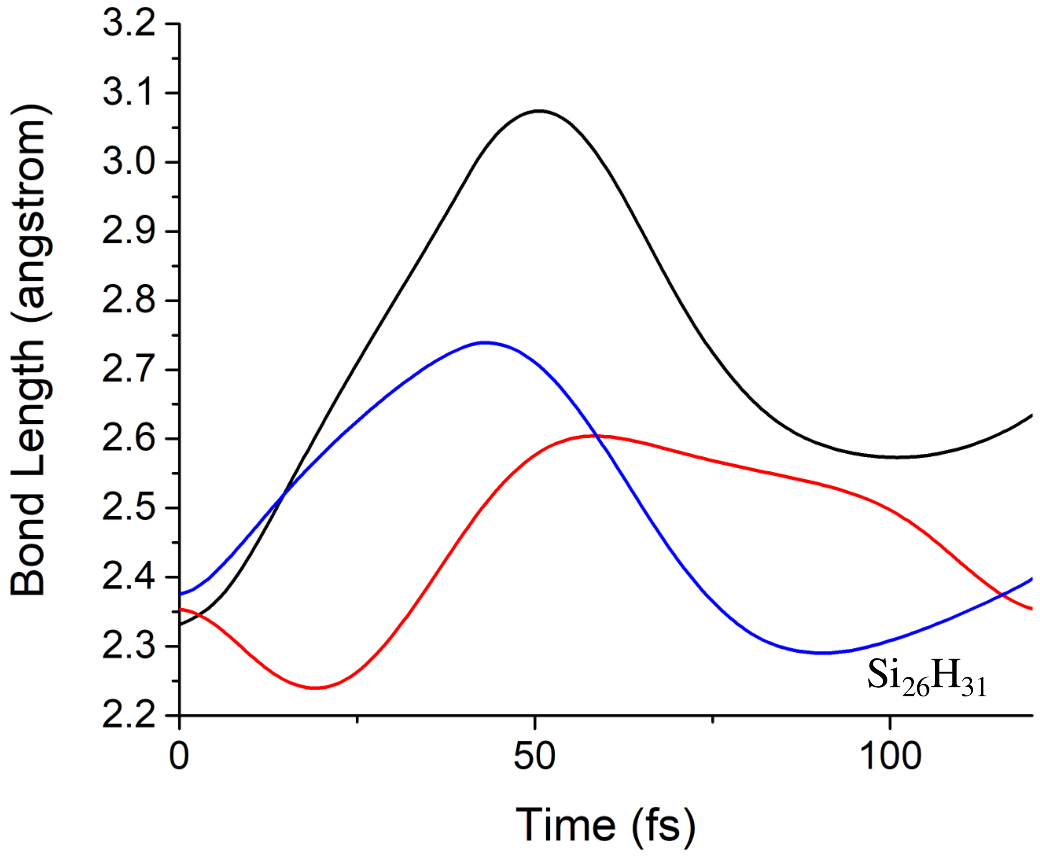

Figure S7. The three Si-Si bond lengths ($R_{Si\text{-}Si}$) as a function of time from the AIMD simulation of $Si_{26}H_{31}$. Each color represents one Si-Si bond length.

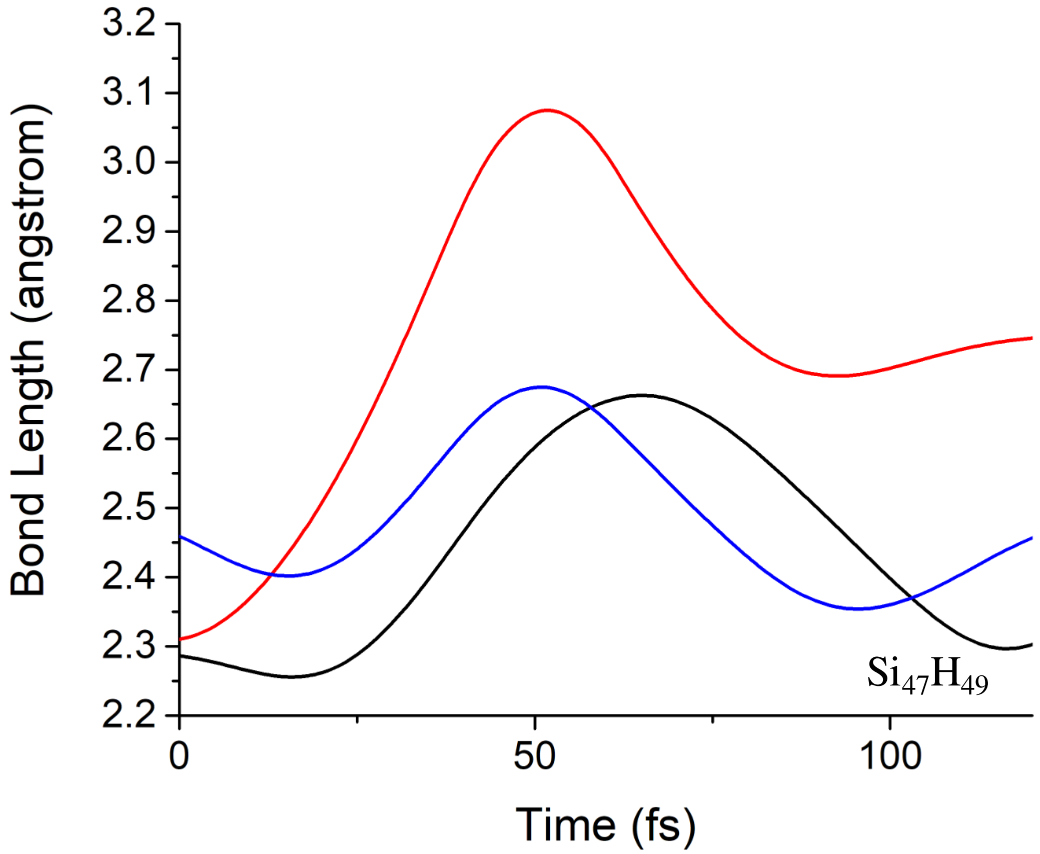

Figure S8. The three Si-Si bond lengths (R_{Si-Si}) as a function of time from the AIMD simulation of $Si_{47}H_{49}$. Each color represents one Si-Si bond length.

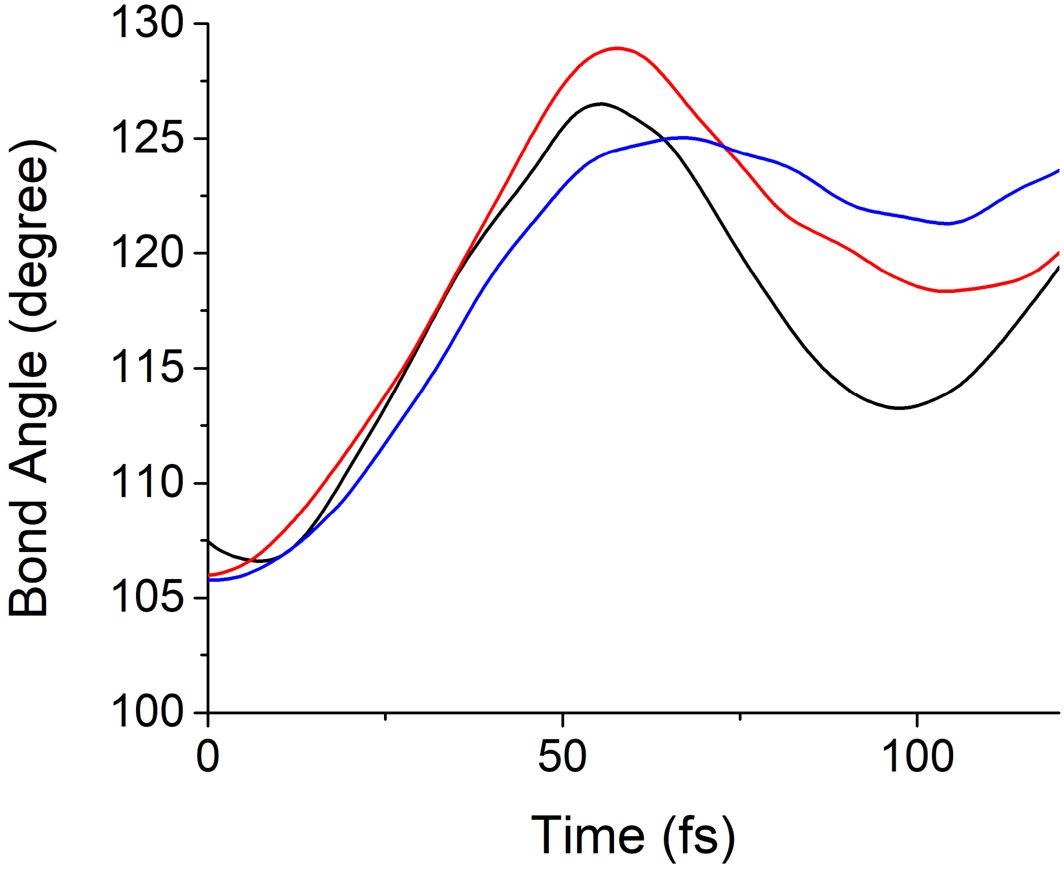

Figure S9. The three Si-Si-Si bond angles (θ, illustrated in Figure 1a) as a function of time from the AIMD calculations of $Si_{10}H_{15}$. Each color represents one Si-Si-Si bond angle.

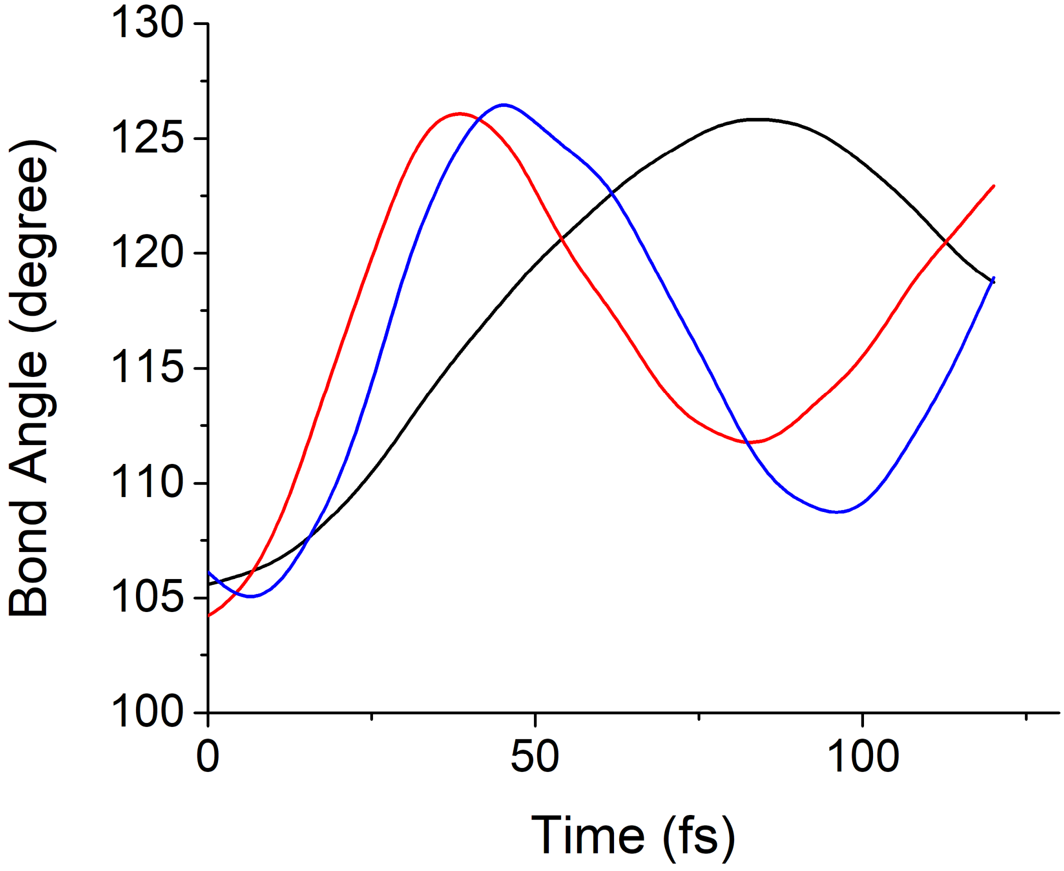

Figure S10. The three Si-Si-Si bond angles (θ, illustrated in Figure 1a) as a function of time from the AIMD calculations of $Si_{22}H_{27}$. Each color represents one Si-Si-Si bond angle.

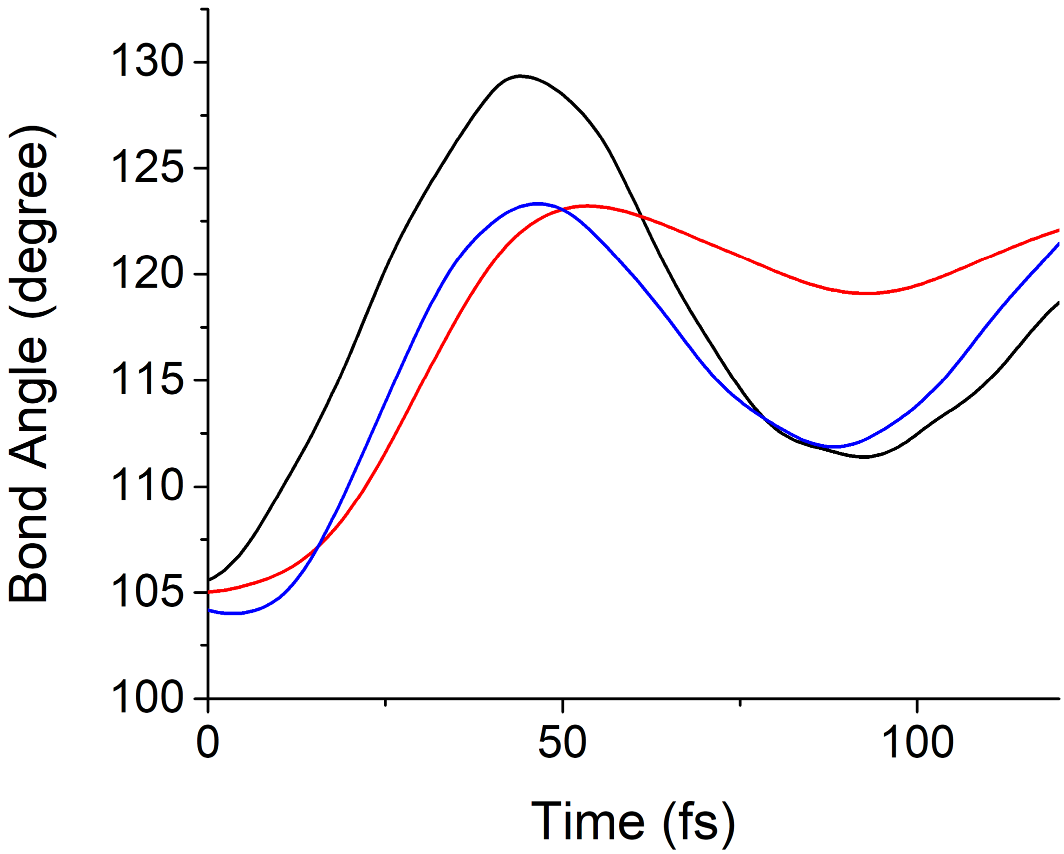

Figure S11. The three Si-Si-Si bond angles (θ, illustrated in Figure 1a) as a function of time from the AIMD calculations of $Si_{26}H_{31}$.

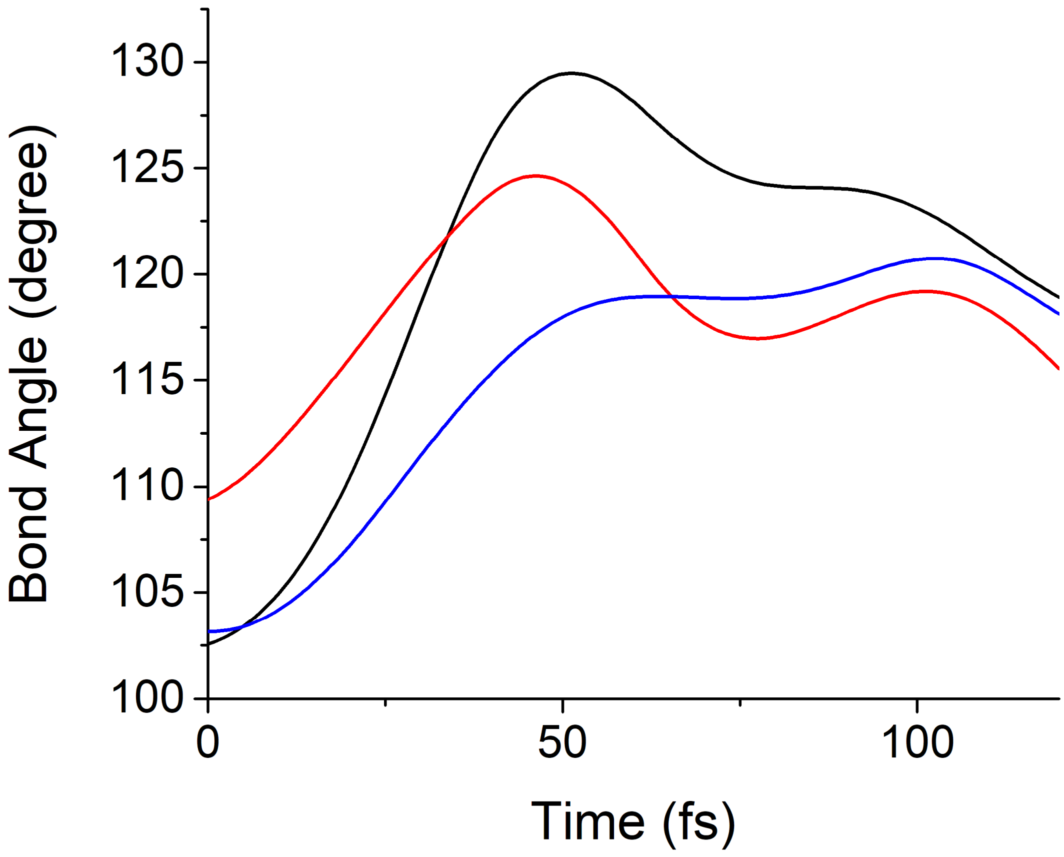

Figure S12. The three Si-Si-Si bond angles (θ, illustrated in Figure 1a) as a function of time from the AIMD calculations of $Si_{47}H_{49}$. Each color represents one Si-Si-Si bond angle.

Supplementary References

1. Granucci, G.; Persico, M.; Toniolo, A., Direct semiclassical simulation of photochemical processes with semiempirical wave functions. *J Chem Phys* **2001,** *114* (24), 10608-10615.
2. Slavicek, P.; Martinez, T. J., Ab initio floating occupation molecular orbital-complete active space configuration interaction: An efficient approximation to CASSCF. *J Chem Phys* **2010,** *132* (23).
3. Hohenstein, E. G.; Bouduban, M. E. F.; Song, C. C.; Luehr, N.; Ufimtsev, I. S.; Martinez, T. J., Analytic first derivatives of floating occupation molecular orbital-complete active space configuration interaction on graphical processing units. *J Chem Phys* **2015,** *143* (1).
4. Wadt, W. R.; Hay, P. J., Abinitio Effective Core Potentials for Molecular Calculations - Potentials for Main Group Elements Na to Bi. *J Chem Phys* **1985,** *82* (1), 284-298.
5. Ufimtsev, I. S.; Martinez, T. J., Graphical processing units for quantum chemistry. *Comput. Sci. Eng.* **2008,** *10* (6), 26-34.
6. Ufimtsev, I. S.; Martinez, T. J., Quantum chemistry on graphical processing units. 1. Strategies for two-electron integral evaluation. *J Chem Theory Comput* **2008,** *4* (2), 222-231.
7. Ufimtsev, I. S.; Martinez, T. J., Quantum Chemistry on Graphical Processing Units. 3. Analytical Energy Gradients, Geometry Optimization, and First Principles Molecular Dynamics. *J Chem Theory Comput* **2009,** *5* (10), 2619-2628.
8. Fales, B. S.; Levine, B. G., Nanoscale Multireference Quantum Chemistry: Full Configuration Interaction on Graphical Processing Units. *J Chem Theory Comput* **2015,** *11* (10), 4708-4716.
9. Werner, H.-J.; Knowles, P. J., A second order multiconfiguration SCF procedure with optimum convergence. *J Chem Phys* **1985,** *82* (11), 5053-5063.
10. Celani, P.; Werner, H. J., Multireference perturbation theory for large restricted and selected active space reference wave functions. *J Chem Phys* **2000,** *112* (13), 5546-5557.
11. Werner, H. J.; Knowles, P. J.; Knizia, G.; Manby, F. R.; Schutz, M., Molpro: a general-purpose quantum chemistry program package. *Wires Comput Mol Sci* **2012,** *2* (2), 242-253.
12. Schmidt, M. W.; Baldridge, K. K.; Boatz, J. A.; Elbert, S. T.; Gordon, M. S.; Jensen, J. H.; Koseki, S.; Matsunaga, N.; Nguyen, K. A.; Su, S.; Windus, T. L.; Dupuis, M.; Montgomery, J. A., General Atomic and Molecular Electronic Structure System. *J. Comput. Chem.* **1993,** *14*, 1347-1363.
13. Gour, J. R.; Piecuch, P.; Wloch, M., Active-space equation-of-motion coupled-cluster methods for excited states of radicals and other open-shell systems: EA-EOMCCSDt and IP-EOMCCSDt. *J. Chem. Phys.* **2005,** *123*, 134113.
14. Gour, J. R.; Piecuch, P., Efficient formulation and computer implementation of the active-space electron-attached and ionized equation-of-motion coupled-cluster methods. *J. Chem. Phys.* **2006,** *125*, 234107.
15. Levine, B. G.; Coe, J. D.; Martinez, T. J., Optimizing conical intersections without derivative coupling vectors: Application to multistate multireference second-order perturbation theory (MS-CASPT2). *J Phys Chem B* **2008,** *112* (2), 405-413.

Molecular Geometries and Absolute Energies

All geometries are in angstrom and absolute energies are in Hartree.

##
Franck-Condon Geometry and Energies
##

Si10H15

Si	-0.5174205979	-0.8348349814	-0.7170299444
Si	1.8349944728	-0.8348264311	-0.7034572298
Si	-1.2975571211	1.2346984879	0.0845084533
Si	-1.3052009638	-2.5548459701	0.6728075910
Si	2.6315781484	-0.4801811161	1.4813624363
Si	-0.5177122134	1.6008056015	2.2734868246
Si	-0.4846934236	-2.1343753473	2.8284603389
Si	1.8346837138	1.5893845329	2.2660259352
Si	-1.3047721410	-0.1085476518	3.6768972784
Si	1.8582635743	-2.1980831303	2.8819602955
H	-0.9982771294	-1.0362895976	-2.0995241051
H	2.3315062053	0.2372717958	-1.5870674988
H	2.3323357458	-2.1296624310	-1.2075209264
H	-0.8158399009	2.3161803939	-0.7958276067
H	-2.7733368421	1.2426787832	0.0773677090
H	-0.8177833037	-3.8633496961	0.1999671071
H	-2.7792581411	-2.5648306177	0.6939505559
H	-0.9987009985	2.9137881883	2.7507928354
H	4.1089148884	-0.4613933574	1.4662466180
H	2.3329439541	-3.5078878963	2.3998373351
H	2.3309456347	-1.9888831636	4.2626165112
H	2.3362682403	1.8278307131	3.6333053374
H	2.3268252019	2.6684318023	1.3883697280
H	-2.7788325440	-0.1308235029	3.6830320283
H	-0.8167544595	0.0893645920	5.0538623924

FOMO-CASCI/LANL2DZ
Energy (D0) = -46.18657640
Energy (D1) = -46.03132021

CASPT2/LANL2DZ
Energy (D0) = -46.291727858657
Energy (D1) = -46.143455745075

IP-EOMCCSD/6-31G**
Energy (D0) = -2898.991769
Energy (D1) = -2898.876997

Si22H27

Si	-0.3972569683	-0.5830501301	-0.8321377189
Si	1.9547937034	-0.5351843517	-0.9261372969
Si	-1.2673419317	1.4207876463	0.0460120336
Si	-1.0925993707	-2.3994500329	0.5183564137
Si	2.7958091258	-0.1644944637	1.2417357013
Si	-0.3752694178	1.7597885391	2.1984031749
Si	-0.1842263064	-1.9641860638	2.6552086261
Si	1.9772606786	1.8458715325	2.1539087435
Si	-1.0702013941	0.0005215740	3.6226425378
Si	2.1782576455	-1.9705589033	2.6428534318
Si	2.7795135709	-2.5536488064	-1.7967200647
Si	-3.6115798856	1.3295809584	0.1346450351
Si	-0.2244978466	-4.4218911388	-0.3137364596
Si	-3.4407387476	-2.4674922803	0.6496570015
Si	2.9663271290	-4.0003233853	1.7513439153
Si	2.9839993170	-1.6272021648	4.8265803683
Si	2.8285758589	2.1711593733	4.3176421018
Si	-3.4187750187	-0.1309944319	3.6768518851
Si	-0.1810162833	0.2952618485	5.7809228431
Si	-4.2716600504	-0.4512847041	1.5148443519
Si	2.1615665176	0.3909193270	5.6953479926
Si	2.1178154977	-4.3296505758	-0.4110542982
H	-0.9337269855	-0.7896112302	-2.1955426474
H	2.3691472072	0.5899615504	-1.7909698734
H	-0.8415282220	2.5392852707	-0.8219118306
H	-0.8977627692	3.0310669206	2.7461811431
H	4.2733117579	-0.1072635696	1.1868895734
H	2.3912495084	2.9635134242	1.2792119398
H	-3.9472753343	1.1236982662	4.2472869753
H	-3.8212391577	-1.2485943134	4.5520456397
H	-3.8462215043	-3.5953227101	1.5100842802
H	-3.9802870975	-2.6951354357	-0.7054811742
H	-4.1531920071	1.1338546260	-1.2239303759
H	-4.1379983402	2.5986143590	0.6729439203
H	-5.7471969680	-0.5132427112	1.5733383063
H	4.3019464522	2.2360897950	4.2671192792

H	2.3201495689	3.4431803692	4.8659126820
H	-0.6127148144	-0.8236447730	6.6402960867
H	-0.7170649358	1.5506740166	6.3428263104
H	2.6949058479	0.5920344669	7.0590176458
H	4.4592270578	-1.5942712095	4.7840164407
H	2.5658146727	-2.7557007535	5.6801035554
H	2.5562723179	-5.1101149082	2.6328987217
H	4.4413386301	-3.9540895487	1.7126277108
H	-0.7772667381	-4.6525748767	-1.6630173745
H	-0.6526344611	-5.5288472442	0.5627333459
H	2.2482628661	-2.7601367084	-3.1579086455
H	2.6373504254	-5.6026008335	-0.9535717420
H	4.2524071997	-2.5002016047	-1.8694102124

FOMO-CASCI/LANL2DZ
Energy (D0) = -98.27824830
Energy (D1) = -98.12211957

Si26H31

Si	-0.4398719991	-0.7667028184	-0.7199895311
Si	1.9169358250	-0.7102511493	-0.8312161401
Si	-1.2842117817	1.2742724470	0.1134434767
Si	-1.1519899237	-2.5663199225	0.6420641284
Si	2.7467564604	-0.3525062221	1.3493626084
Si	-0.4030484240	1.6007804485	2.2788258343
Si	-0.2411305565	-2.1134912524	2.7735430173
Si	1.9549530372	1.6951550095	2.2168084688
Si	-1.1143735472	-0.1348854308	3.7214863010
Si	2.1205673899	-2.1406940490	2.7673489807
Si	2.5808280743	1.0741374168	-2.2171839705
Si	2.7206385134	-2.7580946669	-1.6618975200
Si	-0.5758336726	3.0366740558	-1.2791141000
Si	-3.6319488280	1.1820443817	0.2162064221
Si	-0.2954349561	-4.6001904717	-0.1743329304
Si	-3.5014485152	-2.6102368358	0.7637577222
Si	2.8953657268	-4.1835792702	1.8939246403
Si	2.9336981691	-1.7727609553	4.9446198572
Si	2.7933177311	2.0180727279	4.3902694973
Si	2.6249274101	3.4471592811	0.7924887629
Si	-3.4640844554	-0.2442985623	3.7712029892
Si	-0.2241290923	0.1787678782	5.8764729334
Si	-4.3127756716	-0.5794774241	1.6108681831

Si	2.1180184114	0.2574945304	5.7888243180
Si	2.0465897874	-4.5213280144	-0.2660075568
Si	1.7682817395	3.1040968674	-1.3651261280
H	-0.9710306912	-0.9743469029	-2.0862548834
H	-0.9125347485	2.8871119676	2.8058286964
H	4.2250326162	-0.2987073289	1.2882133424
H	-3.9815046875	1.0217478641	4.3266353671
H	-3.8782921805	-1.3483119622	4.6582253502
H	-3.9239118392	-3.7293332623	1.6276877574
H	-4.0350424388	-2.8398208301	-0.5934380726
H	-4.1745620243	0.9810230975	-1.1417621168
H	-4.1499058706	2.4610358238	0.7401823545
H	-5.7892884662	-0.6267279777	1.6662030424
H	4.2673825755	2.0803816423	4.3433956334
H	2.2896985737	3.2989120615	4.9235788132
H	-0.6650489031	-0.9268836631	6.7483800987
H	-0.7519640874	1.4446426595	6.4226048641
H	2.6526019450	0.4719867603	7.1502497667
H	4.4087895903	-1.7412117524	4.8934142895
H	2.5199654199	-2.8906287069	5.8143326491
H	2.4749943262	-5.2824727349	2.7844520881
H	4.3707110737	-4.1490727793	1.8578148437
H	-0.8470104930	-4.8355329683	-1.5231914466
H	-0.7338434828	-5.6984209044	0.7083249666
H	2.1950252878	-2.9729417343	-3.0243594431
H	2.5597543707	-5.8007898857	-0.8000298153
H	4.1941915726	-2.7136854510	-1.7327245565
H	2.0535348997	0.8514849939	-3.5776806851
H	4.0546489524	1.1107737335	-2.2886280827
H	4.0994803269	3.4969360510	0.7470832498
H	2.1324462611	4.7286577853	1.3346293894
H	-1.0982839833	4.3110636678	-0.7480806102
H	-1.1253410139	2.8371986564	-2.6344226252
H	2.1962402666	4.2121840809	-2.2460944904

FOMO-CASCI/LANL2DZ
Energy (D0) = -115.64864705
Energy (D1) = -115.49108483

$Si_{47}H_{49}$

Si	-0.4818420248	-0.8450923780	-0.7475587158
Si	1.8949966387	-0.7996570386	-0.8162480058

Si	-1.3303573489	1.1989091972	0.1229132491
Si	-1.1755107650	-2.6314669539	0.6580713694
Si	2.7614439979	-0.4119190912	1.3642498020
Si	-0.4519139512	1.5792950376	2.3000190220
Si	-0.2666834627	-2.1565037493	2.7818217331
Si	1.9249267731	1.6337651392	2.2426335061
Si	-1.1507491115	-0.1861076156	3.7293841351
Si	2.0914212410	-2.1910813469	2.7903429621
Si	2.5944697213	0.9792177906	-2.2208695306
Si	2.6807903266	-2.8867062136	-1.6183781784
Si	-0.6396160161	2.9597268145	-1.3085314684
Si	-3.6934319714	1.0790379800	0.2576922038
Si	-0.3592695126	-4.6827916917	-0.1669116664
Si	-3.5194482202	-2.7278756784	0.8443536220
Si	2.8187629272	-4.2757194561	1.9759099962
Si	2.9301101245	-1.8520950816	4.9666858546
Si	2.7611918722	1.9254265007	4.4408754043
Si	2.5933201778	3.4148528825	0.8257341579
Si	-3.5040148649	-0.2787133612	3.8207359208
Si	-0.2819005257	0.0623623586	5.9022623346
Si	-4.3339422246	-0.6953247292	1.6627258444
Si	2.0523344301	0.1543695273	5.8169624287
Si	1.9890879841	-4.6167839264	-0.1827316245
Si	1.7198854433	2.9780875672	-1.3223194444
Si	-1.2293428774	-1.2217303113	-2.9608951612
Si	-1.3483726738	3.6343239864	3.0567823960
Si	5.1213861172	-0.2397435357	1.3416418125
Si	-4.3098523751	1.7613445134	4.6729575549
Si	-4.6890509186	3.0164105966	1.1418196883
Si	5.1086338144	1.9596228770	4.5621460617
Si	5.2805681378	-1.7903940128	4.8715997533
Si	-1.2243641113	-5.0282761474	-2.3278736369
Si	1.8776437223	-3.4401584042	-3.7572951465
Si	1.7262588436	0.8642730044	-4.4080798826
Si	4.9482378499	1.0368641185	-2.2746888332
Si	4.9242128824	3.5630397462	0.5161719393
Si	1.7495958374	5.4389799618	1.6845076927
Si	-1.2604001518	5.1026231602	-0.5512120553
Si	-1.4934024518	2.5742142430	-3.4692906229
Si	-3.6959958783	3.4970237055	3.2138417689
Si	-0.4661705185	-3.3110225378	-3.7401762309
Si	5.9458367901	0.0225087435	3.5339836191
Si	-0.6022266357	0.5836609780	-4.3350375041
Si	-0.5963768059	5.3900298442	1.6809182656

Si	5.7841121117	1.4765960150	-0.1266699457
H	-3.8859362855	-1.3863572271	4.7222133972
H	-3.8751739213	-3.8373381830	1.7492600550
H	-4.1024051055	-2.9992501783	-0.4831471239
H	-4.2257575946	0.8246175845	-1.0975283260
H	-5.8114485808	-0.7338762132	1.7199904631
H	2.2201947541	3.1900623677	4.9819150730
H	-0.7316548092	-1.0779557287	6.7230349423
H	-0.8074568981	1.3055273298	6.4973343607
H	2.5953744091	0.3739229220	7.1751601098
H	2.4903655432	-2.9835896413	5.8103323133
H	2.3456272198	-5.3308164031	2.8918611470
H	4.2934703939	-4.3011898528	1.9519397105
H	-0.8198921900	-5.7579406416	0.7371122698
H	2.4978651041	-5.8980430385	-0.7187408229
H	4.1583325695	-2.8521895154	-1.6392676432
H	2.1484598895	4.0858135310	-2.2077360361
H	5.8188261508	-1.6159927180	6.2346614169
H	5.7881138508	-3.0612517576	4.3228916287
H	7.4217518662	0.0823595037	3.4814031858
H	5.6723371414	3.1793836881	3.9643513991
H	5.4854232127	1.9284875125	5.9895040901
H	7.2611244374	1.5332093712	-0.1601782368
H	5.1514627665	4.5188882624	-0.5866263415
H	5.5993602751	4.0868526713	1.7143465869
H	2.2204062097	6.5560069793	0.8417857946
H	2.2662621744	5.6382663286	3.0518565379
H	-1.1300152265	6.6670161657	2.2009631224
H	-2.7008934004	5.3455417253	-0.7285938053
H	-0.5298639645	6.0806816764	-1.3827483960
H	5.3854025508	2.1001812319	-3.2007537064
H	5.4533967566	-0.2546809505	-2.7779471062
H	2.0179429905	2.1596918784	-5.0549396069
H	2.3796383149	-0.1920363209	-5.1973426781
H	-1.1206506992	0.3484369495	-5.6995239532
H	-2.9654398079	2.5027405608	-3.4032652102
H	-1.1211729008	3.7027132892	-4.3453394285
H	-2.7078395095	-1.2758026474	-2.9411079642
H	-0.9888588572	-3.5273706027	-5.1057061256
H	-0.7499465958	-6.3283618900	-2.8404151937
H	-2.6976049557	-5.0513664116	-2.2743048301
H	5.6675240594	-1.5113586086	0.8181947097
H	2.4616213138	-2.5854439499	-4.8020046494
H	2.2644308634	-4.8378826380	-4.0348252662

H	-4.2125441458	4.7821908724	3.7297934077
H	-5.7812647245	1.7014364729	4.7701212832
H	-3.7568532536	1.9887112059	6.0206386615
H	-6.1186061319	2.7239426360	1.3678727238
H	-4.5935550529	4.1551233989	0.2159800954
H	-0.8325825741	3.8833259734	4.4210475184

FOMO-CASCI/LANL2DZ
Energy (D0) = -205.09486192
Energy (D1) = -204.94811766

Si72H63

Si	0.0142241203	-0.0252846567	0.0768646407
Si	0.0346997444	-0.0401227583	2.4414756915
Si	2.2575291124	0.0123854838	-0.7170080344
Si	2.2269845101	0.0275667081	-3.0942123496
Si	-1.1397905465	-1.9564352762	-0.6992361571
Si	-1.1169672063	1.9175074904	-0.7219432065
Si	-1.1576391126	-1.9337091445	-3.0764343523
Si	-1.1324828590	1.9214860268	-3.1022953316
Si	-0.0089272882	-3.9048106254	0.0565840655
Si	3.3922990567	-1.9333970945	0.0397311433
Si	0.1940503141	-3.9783251531	2.4053931311
Si	3.3725782305	-2.1323839650	2.3904206130
Si	1.1645852039	-1.9837872048	3.1851727340
Si	2.1719975613	-3.7553835232	-0.8169293028
Si	-2.2920927412	0.0014049753	-3.8884208992
Si	1.1039942636	1.9690430258	-3.9065620857
Si	1.0810055341	-1.9030507914	-3.8870353468
Si	-2.2562654975	3.8547204996	-3.8945861082
Si	-2.2523561113	3.8298838715	-6.2620365959
Si	-3.2746325565	1.9127465058	-7.1625716490
Si	-0.0873905138	3.7590490879	-7.1778926271
Si	-2.2720068559	-0.0211748796	-6.2610696976
Si	1.0957859215	1.9290465522	-6.2793755974
Si	-0.0320338637	-0.0131251069	-6.9886978399
Si	2.2152395250	-3.8524750271	-3.1670272271
Si	1.0921088668	-1.9443224955	-6.2520793138
Si	4.4427814737	-3.8277688209	-3.9601626326
Si	1.0801913498	-5.7764320480	-3.9430256700
Si	-0.0277910630	-3.8859502178	-7.0011095015
Si	3.3277638978	-1.9428613762	-7.0186636634

Si	1.1244517982	-5.8027701264	-6.2915397414
Si	4.4240250368	-3.8917365542	-6.3083633081
Si	-2.1977898930	-0.0016194395	3.2279762350
Si	1.1169638634	1.9200254425	3.2102560129
Si	-3.3323125511	1.9406687411	0.0998412176
Si	-0.0299565104	3.8550378699	0.0823035009
Si	-4.4656082230	3.8534117935	-3.0446885343
Si	-1.1513717485	5.7750655685	-3.0612606269
Si	-1.1762957101	5.7675193883	-0.7060102251
Si	-0.0357924735	3.8276198904	2.4463205355
Si	-3.2877847392	1.9422087721	2.4634526806
Si	-4.4280516160	3.8823805864	-0.6896083457
Si	-2.2404689841	3.8899128591	3.2469016364
Si	-3.3632035610	5.8002105993	0.1424363872
Si	-3.3561099228	5.8082547919	2.4877000092
Si	-3.2346696447	-1.9598306240	-7.1869488927
Si	-4.5119858403	0.0107269519	-3.0502515018
Si	-2.2855978847	-3.8606541916	-3.8567065167
Si	-2.2513791794	-3.8642965448	-6.2236791040
Si	-0.8955075372	-5.9242935790	-0.7746374599
Si	-1.1208204174	-5.7807103101	-3.1080003619
Si	-3.3716697116	-1.9319512400	0.1174679077
Si	-4.4188517815	0.0157331836	-0.6965260507
Si	-5.7050631671	-1.9219747662	-3.6746701917
Si	-4.6082953956	-3.7695972942	-0.6839547211
Si	-4.5055505891	-3.8415351567	-3.0323898522
Si	3.3305469068	-5.8146847986	-7.0861842545
Si	3.3516360758	1.9656919225	0.0810170439
Si	2.2048992291	3.9044161170	-3.0867247941
Si	2.1740340752	3.8374616935	-0.7321670002
Si	5.5467840461	-1.9157290608	-3.1431640868
Si	4.4204942138	0.0008111951	-6.2597663805
Si	4.4530841503	0.0452328946	-3.8933023715
Si	5.5530377927	2.1292009520	-0.7417784001
Si	4.4668496462	3.9741396258	-3.7395437411
Si	5.5449466650	1.9867753683	-3.0894829012
Si	3.3181220300	2.1616378953	2.4267966383
Si	-3.5049464308	-1.7968665710	2.4637278514
Si	-5.7141847553	1.9520801125	-3.6304626877
Si	1.1144103948	5.9111350362	-3.6642925358
Si	5.5792623203	-2.1661673277	-0.8079868935
Si	3.2457820888	1.7901203204	-7.2287832961
H	3.3294258772	-5.8252786239	-8.5612795450
H	4.0307907851	-7.0197495786	-6.6024741603

H	0.4050367645	-7.0063137169	-6.7596789923
H	5.8219188057	-3.8683799275	-6.7884697769
H	5.1240361038	-5.0278012291	-3.4280182341
H	3.2963233770	-1.9090342418	-8.4975059835
H	1.7881197862	-6.9612758834	-3.4116902747
H	-0.0536240787	-3.8489242016	-8.4800230480
H	5.8167341193	-0.0151380906	-6.7475452777
H	6.9331944217	-1.9182992981	-3.6588470167
H	-1.8125536084	-6.9866563461	-3.6131456451
H	-2.9350844591	-5.0857433756	-6.7016869713
H	-2.1557407259	-6.2670192120	-0.0989349870
H	0.0871465362	-6.9843394534	-0.4760635219
H	6.0213639124	-3.5406422807	-0.5015065070
H	6.5032141846	-1.2316573450	-0.1481688338
H	6.9356944424	1.9835493131	-3.5927156454
H	3.9780637396	3.0617076432	-7.1306394516
H	3.0675395064	1.4785931668	-8.6614519288
H	-0.0411831528	-0.0134110108	-8.4687141404
H	-4.6999164117	-1.9622589549	-7.0598473957
H	-2.9039391886	-1.9676912736	-8.6263084957
H	-5.1969421500	-5.0532431888	-3.5235604103
H	-4.7423551943	5.8140120304	2.9928249244
H	-2.6674336406	7.0156105574	2.9826317342
H	-4.0607410847	6.9988572255	-0.3698020210
H	-2.2171476938	3.8637572827	4.7248940991
H	0.6998669372	5.0226699930	2.9150575824
H	-0.4382281706	6.9596104243	-0.2336173924
H	-5.8260067600	3.8380805016	-0.2071350442
H	-4.6871330116	1.8988753859	2.9420646976
H	-5.1509848247	5.0727391089	-3.5265212821
H	-1.8734201042	6.9726348975	-3.5439614291
H	1.1062881603	1.9122852253	4.6893452109
H	-2.1725010493	0.0108612683	4.7068002061
H	-5.8108147169	0.0085511911	-0.1934183682
H	1.1762166089	-1.9931735808	4.6641121260
H	2.8760695207	5.0435095895	-0.2390188285
H	-2.9545763086	5.0395554241	-6.7433972762
H	-6.9459872919	1.9565329808	-2.8154325006
H	-6.1104039164	1.9502023130	-5.0469435022
H	-3.1458340514	-3.0703875536	3.1066082790
H	-4.9125327652	-1.5013802083	2.7997225986
H	3.7592771113	3.5334495751	2.7510222232
H	4.2523940966	1.2240063210	3.0689281187
H	1.3031452370	6.2513511597	-5.0826892336

H	1.7300593092	6.9826652672	-2.8553742254
H	-6.9727762757	-1.9227277101	-2.9166639727
H	-6.0367596186	-1.9134730067	-5.1072716468
H	-6.0192835367	-3.5491531073	-0.3072968675
H	-4.1663172785	-5.0244648727	-0.0568752474
H	-1.1082197360	-4.2653518755	3.0242925262
H	1.1108720070	-5.0877008497	2.7332951953
H	4.2684436566	-1.1365254850	2.9963154923
H	3.8878706450	-3.4748230174	2.7238099150
H	6.0576241624	3.4696070179	-0.3803899101
H	6.4348812122	1.1326402387	-0.1150591964
H	4.6026455996	4.2472009580	-5.1780569907
H	5.1086291622	5.0838120453	-3.0059758344
H	-0.2259383858	3.5249706989	-8.6295609701
H	-4.7322154567	1.8993213135	-6.9687363699
H	0.6525473782	5.0161682626	-6.9926420676
H	-3.0110230539	1.9082580693	-8.6158982183

FOMO-CASCI/LANL2DZ
Energy (D0) = -307.38701171
Energy (D1) = -307.24001025

##

Conical Intersections Geometry and Energies

##

Si10H15

Si	-0.6996444474	-0.3599381537	-2.1600644987
Si	1.6411965369	-0.2588087061	-2.2271954014
Si	-1.5047575705	1.7029830274	-1.3762412317
Si	-1.5233684204	-2.0615895409	-0.7616660576
Si	2.4129366370	0.1831194584	-0.0586584156
Si	-0.7381650373	2.0709205294	0.8160787228
Si	-1.3230487932	-2.0352563340	1.6590115999
Si	1.6012917406	2.2009342602	0.8182538788
Si	-1.4217361747	0.3364398579	2.2532644160
Si	1.5498842112	-1.5193595133	1.2939760326
H	-1.1894452595	-0.5829778082	-3.5371368266
H	2.0895212220	0.8152258606	-3.1320477228
H	2.2290290056	-1.5362410419	-2.6731123834
H	-1.0177372499	2.7898386377	-2.2482359432

H	-2.9802749068	1.7071580085	-1.3978971508
H	-1.1753279097	-3.3868367719	-1.3132995964
H	-2.9952022759	-1.9187743788	-0.8350903745
H	-1.2909804203	3.3502507867	1.3109325131
H	3.8865169352	0.0916864651	0.0251953578
H	1.7288052142	-2.8982183979	0.8194759763
H	1.7889920415	-1.4145255534	2.7392467162
H	2.1042473155	2.3333168542	2.1984914520
H	2.0977330926	3.3320544937	0.0143491891
H	-2.8911155547	0.4550926922	2.3712081909
H	-0.8342181199	0.5584675196	3.5909461335

FOMO-CASCI/LANL2DZ
Energy = -46.08852232

CASPT2/LANL2DZ//FOMO-CASCI/LANL2DZ
Energy (D0) = -46.208359193351
Energy (D1) = -46.194010061551

Si22H27

Si	-0.5940904555	0.3616540930	-2.6805104047
Si	1.7550557026	0.3451103256	-2.7684170382
Si	-1.5027579220	2.3288649207	-1.7751423935
Si	-1.2363980971	-1.3821862538	-1.2579914285
Si	2.5206230408	0.6194792255	-0.5644464069
Si	-0.6223138325	2.5199713053	0.3938666098
Si	-0.4579089825	-1.5426175774	1.5170891127
Si	1.7245842491	2.6301537011	0.3484312496
Si	-1.3506481163	0.7376816209	1.7665783944
Si	1.8628161468	-1.2060408196	0.7832590069
Si	2.5461552059	-1.6991291200	-3.6242685779
Si	-3.8514324626	2.2437742855	-1.6661730014
Si	-0.4247163217	-3.5030297102	-1.9302512060
Si	-3.5791973509	-1.5494718203	-0.9617691224
Si	2.8249455405	-3.1520779670	-0.0992648329
Si	2.7487814160	-0.8237789737	2.9529312344
Si	2.5349274167	2.9812440810	2.5229366042
Si	-3.6910275005	0.7951223070	1.9014831982
Si	-0.4546834633	1.1098872702	3.9372683172
Si	-4.5159400527	0.4813907617	-0.2645026140
Si	1.8943416151	1.1669942115	3.8653316176
Si	1.8987730682	-3.4713343033	-2.2243944500

H	-1.1738164171	0.0618354369	-4.0103226054
H	2.2233553657	1.4609977905	-3.6174114922
H	-1.0820628018	3.4851591718	-2.5921224151
H	-1.1311402304	3.7742085309	0.9937682765
H	4.0018056655	0.6405725429	-0.5772747866
H	2.1293714308	3.7505192376	-0.5264611276
H	-4.1254793892	2.0894975839	2.4583237127
H	-4.1668349098	-0.2920070314	2.7764718682
H	-3.8383227001	-2.5917138873	0.0476790966
H	-4.1193916952	-2.0109926956	-2.2573476752
H	-4.4356372698	2.0805794118	-3.0109482601
H	-4.3364463599	3.5202373987	-1.1044861996
H	-5.9877693936	0.3535617047	-0.2719996540
H	4.0030027383	3.1287090971	2.5078782075
H	1.9425424050	4.2228044921	3.0585507558
H	-0.9253699345	0.0416400900	4.8396980552
H	-0.9556362606	2.4037068863	4.4380001433
H	2.4287965302	1.3521745496	5.2300637492
H	4.2176666815	-0.7304585330	2.8561902564
H	2.4024285512	-1.9715876941	3.8136684006
H	2.5335709937	-4.3015705297	0.7756058785
H	4.2865059047	-2.9763295786	-0.1977124321
H	-1.1439095982	-3.8178919403	-3.1822540359
H	-0.8091058287	-4.5011490261	-0.9158392080
H	2.0444184474	-1.9098346826	-4.9950265229
H	2.3212195488	-4.7585025460	-2.8135108121
H	4.0213152921	-1.6561013422	-3.6660899702

FOMO-CASCI/LANL2DZ
Energy = -98.18548068

Si26H31

Si	-0.8709514730	-0.3598917130	-2.0828395194
Si	1.4823861941	-0.2789482831	-2.2262195021
Si	-1.6831631158	1.7131541168	-1.3094300428
Si	-1.5977718188	-2.1626259592	-0.7260862307
Si	2.2821092473	0.0710462959	-0.0416083340
Si	-0.7651574189	2.0238896069	0.8369561429
Si	-0.7775062319	-2.4802385376	1.5423288250
Si	1.5822403453	2.1618023327	0.7837201548
Si	-1.3487698321	0.2169484084	2.1926419629
Si	1.5922199337	-1.6737971224	1.4009136994

Si	2.1646156980	1.5112801588	-3.6026943390
Si	2.2969119331	-2.3259796636	-3.0571230367
Si	-0.9842011486	3.4975404496	-2.6770488297
Si	-4.0307472157	1.5972344900	-1.1998517686
Si	-0.7713897166	-4.1491739554	-1.7161888286
Si	-3.9439556631	-2.2201736091	-0.7473688103
Si	2.3617564939	-3.7438706793	0.4895905362
Si	2.6102222265	-1.3463988678	3.4886051800
Si	2.4636911274	2.4588818875	2.9486233415
Si	2.2114977237	3.9375761211	-0.6283823029
Si	-3.7076080092	-0.0511014420	2.2855585972
Si	-0.5015946718	0.4729089195	4.3859688209
Si	-4.6845264202	-0.1965039420	0.1607605823
Si	1.8297625042	0.6823955693	4.3450127862
Si	1.5742804039	-4.0905601260	-1.6929007380
Si	1.3591846132	3.5565108056	-2.7795380134
H	-1.4073674926	-0.5684896309	-3.4496704212
H	-1.3490389563	3.2289711340	1.4692127677
H	3.7644290172	0.0548104427	-0.0649945724
H	-4.1704624446	1.1502893567	3.0117263703
H	-4.0343093484	-1.2369843982	3.0927944156
H	-4.4178112064	-3.3310711416	0.0992663737
H	-4.4646432358	-2.4064381830	-2.1154486182
H	-4.5419809386	1.3756796591	-2.5674395666
H	-4.5831572180	2.8708142273	-0.7042138547
H	-6.1513746662	-0.1828351849	0.3306144362
H	3.9362662048	2.4941836154	2.8362571721
H	2.0078884660	3.7443755151	3.5105071943
H	-0.9084620468	-0.6879045272	5.1994562432
H	-1.1453619760	1.6853577445	4.9310994383
H	2.3187925948	0.9236854608	5.7190456407
H	4.0740747003	-1.3101987721	3.3012266728
H	2.2631580003	-2.4466056823	4.4043910733
H	1.9224478461	-4.8306326005	1.3810335170
H	3.8377664341	-3.7039342450	0.4879548551
H	-1.2469650110	-4.2642562735	-3.1099497042
H	-1.2601676749	-5.3146501793	-0.9542517928
H	1.8007534500	-2.5184073326	-4.4331022372
H	2.1255415115	-5.3622769010	-2.2051764996
H	3.7718890467	-2.2797622740	-3.0910929885
H	1.6593084830	1.2970602796	-4.9716520939
H	3.6410598188	1.5412274457	-3.6495792725
H	3.6850194558	4.0547529181	-0.6581450301
H	1.6556030870	5.1877645473	-0.0748261669

H	-1.4967916662	4.7544655035	-2.0988753773
H	-1.5549736226	3.3403509844	-4.0286969087
H	1.7878591380	4.6513070455	-3.6752175997

FOMO-CASCI/LANL2DZ
Energy = -115.55608964

Si47H49

Si	-1.0805163359	-1.0841087306	-1.5771394436
Si	1.2730095406	-0.9859890166	-1.7430896817
Si	-1.9594752751	0.9470017532	-0.7123441710
Si	-1.6589471478	-2.8792031405	-0.1364528900
Si	2.0880606584	-0.6022105005	0.4419824348
Si	-1.0984706687	1.3060060935	1.4686582831
Si	-1.3804333890	-2.8005157439	2.3870804849
Si	1.2683286793	1.4243584888	1.3688853256
Si	-1.7771225778	-0.4590647147	2.9127505529
Si	1.3296261654	-2.3642270603	1.7788367191
Si	1.9617901828	0.8030323164	-3.1233441522
Si	2.0464527242	-3.0811023566	-2.5566238023
Si	-1.2934364012	2.7131259106	-2.1499938956
Si	-4.3221979913	0.7942518029	-0.5793705540
Si	-0.9578634906	-4.9268210788	-1.0549409057
Si	-4.0423729610	-2.9647157019	0.0474973477
Si	2.0728757438	-4.4821870866	1.0554050198
Si	2.0727929308	-2.0901249390	4.0184129425
Si	2.1132758642	1.6834184735	3.5736166516
Si	1.9374420562	3.2072371833	-0.0381341679
Si	-4.1441316747	-0.4707314865	3.0172867845
Si	-0.9811062610	-0.0807160889	5.0871890812
Si	-4.9347988066	-0.9608767073	0.8595411802
Si	1.3437084607	-0.0626814944	4.9472014678
Si	1.3816834996	-4.8382216827	-1.1383153100
Si	1.0660892172	2.7744967477	-2.1896031643
Si	-1.8831327919	-1.4559480238	-3.7776353409
Si	-1.9900298926	3.3792715186	2.1963822061
Si	4.4504997352	-0.4894696345	0.4675275650
Si	-4.9733493506	1.5799704030	3.8131647647
Si	-5.3301868663	2.7401201315	0.2670210574
Si	4.4570073061	1.6408014720	3.7311366905
Si	4.4377669585	-2.1416630484	3.9257200319
Si	-1.9192139135	-5.2443957890	-3.1752871020

Si	1.1823430219	-3.6304848912	-4.6768476540
Si	1.0536428443	0.6843412662	-5.2949068780
Si	4.3173762793	0.8901516243	-3.1395463639
Si	4.2763493778	3.3357630484	-0.2907762346
Si	1.0888224525	5.2398163413	0.7928676092
Si	-1.9335359852	4.8526776727	-1.3982258975
Si	-2.1844718619	2.3162024613	-4.2934647126
Si	-4.3399731709	3.2795803135	2.3258884482
Si	-1.1598151429	-3.5402635970	-4.5974557428
Si	5.2293212478	-0.3104764429	2.6811935871
Si	-1.2672353588	0.3455136613	-5.1679846886
Si	-1.2531340657	5.1447482067	0.8263630714
Si	5.1283771411	1.2546600126	-0.9636777148
H	-4.5410739400	-1.5527447654	3.9414566464
H	-4.4132789808	-4.1170622451	0.8835354701
H	-4.5443977790	-3.1928530189	-1.3233510096
H	-4.8489659278	0.5087864537	-1.9299454459
H	-6.4110597236	-1.0611176823	0.8637645288
H	1.6019677579	2.9674342594	4.0976149243
H	-1.4217452843	-1.1851764240	5.9599788476
H	-1.4740269899	1.1945435757	5.6385436616
H	1.9863308135	0.0710480392	6.2723721381
H	1.5854836010	-3.2320282823	4.8181754077
H	1.5128891733	-5.5038430003	1.9570823058
H	3.5439865828	-4.5100082724	1.1674269714
H	-1.3713293053	-5.9978611291	-0.1252088080
H	1.9394899825	-6.1000218844	-1.6724705328
H	3.5230393488	-3.0477836767	-2.6168140070
H	1.4680869818	3.8983849995	-3.0666762460
H	4.9536837759	-2.0575831999	5.3060639554
H	4.8917680998	-3.4094448704	3.3275131468
H	6.7061679720	-0.3313278428	2.6696083416
H	5.0798531411	2.8482242990	3.1655885680
H	4.8032574466	1.5671980584	5.1638241966
H	6.6065763308	1.2946260092	-0.9809404479
H	4.5496874851	4.3150942039	-1.3615471258
H	4.9195002642	3.8184897069	0.9413822324
H	1.5260529822	6.3323746583	-0.0981388439
H	1.6370103274	5.4909909487	2.1384758784
H	-1.8049075597	6.4106298028	1.3535310979
H	-3.3780801353	5.0692193935	-1.5694749264
H	-1.2220214589	5.8330583512	-2.2439817550
H	4.7506917211	2.0135698284	-3.9945777156
H	4.8577461308	-0.3604920813	-3.7019150527

H	1.2881991394	1.9930697710	-5.9379331725
H	1.7155626434	-0.3462410152	-6.1101109085
H	-1.7992275312	0.0898288593	-6.5242289285
H	-3.6487859746	2.1975276122	-4.1788151979
H	-1.8744855797	3.4545300305	-5.1816022029
H	-3.3609887150	-1.4838066585	-3.7390219633
H	-1.7061774496	-3.7383832448	-5.9560181943
H	-1.5130379633	-6.5612865860	-3.7046082800
H	-3.3884394172	-5.2092047319	-3.0705168464
H	4.9740742875	-1.7599180691	-0.0794442084
H	1.7112405500	-2.7498540184	-5.7291302974
H	1.5977190621	-5.0151450158	-4.9778606456
H	-4.8437189582	4.5882517738	2.7933657081
H	-6.4469617364	1.5309177546	3.8902858626
H	-4.4407775950	1.8372424359	5.1631119043
H	-6.7536859424	2.4284330714	0.5069765306
H	-5.2588745496	3.8495503069	-0.6942662775
H	-1.4887048558	3.6433093787	3.5632932730

FOMO-CASCI/LANL2DZ
Energy = -205.00517353

$Si_{72}H_{63}$

Si	0.0076002773	0.0056878681	2.3708277395
Si	0.0388678163	-0.0271752787	4.7433961007
Si	2.2231615770	0.0460193503	1.5502747400
Si	2.2309883884	0.0643150535	-0.8191592070
Si	-1.0660751180	-1.9621135920	1.6143122203
Si	-1.1177450367	1.9420816251	1.5795860257
Si	-1.1044802497	-1.9183219249	-0.7636832829
Si	-1.1208353745	1.9503635050	-0.8034979572
Si	0.1301609942	-3.8721305426	2.3984874764
Si	3.2514668906	-1.9332589739	2.2374923626
Si	0.1938841260	-3.9748777120	4.7475493911
Si	3.2965035309	-2.2402336536	4.5774371711
Si	1.1580409709	-1.9724038170	5.4960694492
Si	2.3655890295	-4.5148827101	1.5266609144
Si	-2.2569459388	0.0077989005	-1.5797008146
Si	1.1153121864	2.0032212908	-1.6160278910
Si	1.1360977731	-1.9045997432	-1.5552269043
Si	-2.2511119269	3.8774231123	-1.5941466582
Si	-2.2501982494	3.8445155187	-3.9618836196
Si	-3.2920693497	1.9173367229	-4.8158434595

Si	-0.0928976178	3.7689470538	-4.8972864592
Si	-2.2405977541	-0.0088411470	-3.9540753312
Si	1.1066941992	1.9557245775	-3.9887394326
Si	-0.0006399813	-0.0063687198	-4.6770408721
Si	2.3185666224	-3.8253030086	-0.8165559723
Si	1.1236974130	-1.9327837569	-3.9235026057
Si	4.4958219790	-3.8050515651	-1.7201730349
Si	1.1355267989	-5.7329503979	-1.6297484031
Si	0.0065071529	-3.8673699020	-4.6944504959
Si	3.3412608519	-1.9179429734	-4.7380920371
Si	1.1533283460	-5.7808705948	-3.9757829612
Si	4.4533693174	-3.8700665055	-4.0619187771
Si	-2.1949866705	0.0001916491	5.5231179376
Si	1.1284092509	1.9269302186	5.5090838101
Si	-3.3322328673	1.9562066057	2.4007970846
Si	-0.0237825128	3.8758716038	2.3893989698
Si	-4.4575359232	3.8719699829	-0.7385149150
Si	-1.1484542182	5.7960154540	-0.7560246118
Si	-1.1731836457	5.7859797714	1.5991457948
Si	-0.0237546411	3.8378079940	4.7535862745
Si	-3.2837909112	1.9457248444	4.7646212878
Si	-4.4248951462	3.8996783235	1.6152457231
Si	-2.2309368513	3.8895113349	5.5538717009
Si	-3.3566633100	5.8107704624	2.4557169506
Si	-3.3302852151	5.8197767295	4.8004680732
Si	-3.2028966949	-1.9481115614	-4.8772995077
Si	-4.4790396219	0.0235634836	-0.7441136282
Si	-2.2451393375	-3.8397815971	-1.5492493921
Si	-2.2134434203	-3.8462197707	-3.9151866920
Si	-0.8836131062	-5.8534195153	1.5537186624
Si	-1.0657385351	-5.7445897805	-0.7845133043
Si	-3.3038699787	-1.9449390977	2.4087992635
Si	-4.3733830466	0.0025995986	1.6076930327
Si	-5.6731813604	-1.8985494286	-1.3957598948
Si	-4.6061115241	-3.7353471865	1.5981350578
Si	-4.4727910760	-3.8151922249	-0.7483541829
Si	3.3527705657	-5.8108167116	-4.7878257736
Si	3.3613180139	1.9594758698	2.3734002379
Si	2.2145542231	3.9343855207	-0.7782135698
Si	2.1840045051	3.8467346744	1.5780379879
Si	5.5596252189	-1.8869396835	-0.8781057869
Si	4.4330166157	0.0269401859	-3.9732774914
Si	4.4654595593	0.0797696298	-1.6059128755
Si	5.5664383491	2.1185873056	1.5530678480

Si	4.4843011713	4.0193135439	-1.4035332562
Si	5.5559306464	2.0161316189	-0.7953483948
Si	3.3374274601	2.0774630361	4.7257763447
Si	-3.4698393347	-1.8138707218	4.7520130667
Si	-5.7067968479	1.9610214077	-1.2849404613
Si	1.1164724177	5.9340999367	-1.3612258894
Si	5.4567684811	-2.2195877210	1.4342453542
Si	3.2727534258	1.8417241656	-4.9073993858
H	3.3462226780	-5.8757488699	-6.2611360495
H	4.0575605465	-6.9974131540	-4.2653154696
H	0.4228055487	-6.9830750383	-4.4318051355
H	5.8422355555	-3.8476807973	-4.5691446044
H	5.2102235349	-4.9867614124	-1.1908601692
H	3.2799795304	-1.8593258373	-6.2154633257
H	1.8291913638	-6.9283667253	-1.1067286987
H	-0.0154167020	-3.8158962458	-6.1729377676
H	5.8327736596	0.0170082342	-4.4530938889
H	6.9723057679	-1.8441731863	-1.3124276253
H	-1.7585878451	-6.9531071087	-1.2812313213
H	-2.9032559171	-5.0686477947	-4.3807009684
H	-2.1844335052	-6.0562979790	2.2059284518
H	-0.0150511567	-6.9901711361	1.9069712129
H	5.8870691918	-3.5932552577	1.7485806756
H	6.3022436936	-1.2959688665	2.2096049735
H	6.9452124135	2.0191709680	-1.3015674417
H	4.0005769785	3.1068692531	-4.7317832803
H	3.1327486158	1.6046783296	-6.3575156110
H	-0.0016345125	-0.0220192205	-6.1575893667
H	-4.6671523728	-1.9458092559	-4.7508424050
H	-2.8772262187	-1.9489619377	-6.3169131662
H	-5.1555624499	-5.0268163064	-1.2509614564
H	-4.7102404438	5.8521220266	5.3196531593
H	-2.6175022376	7.0185907091	5.2819160604
H	-4.0566770432	7.0082452598	1.9460349560
H	-2.2052634557	3.8626155354	7.0317085293
H	0.7059525339	5.0379451000	5.2204600205
H	-0.4382938287	6.9799115442	2.0708599351
H	-5.8250762583	3.8570148611	2.0895292391
H	-4.6828899720	1.9096361080	5.2445886612
H	-5.1430227770	5.0896711229	-1.2222828390
H	-1.8732032858	6.9925601329	-1.2366616102
H	1.1242799976	1.9194311872	6.9886279421
H	-2.1651284030	0.0089185832	7.0020961362
H	-5.7634210639	-0.0330270519	2.1141907802

H	1.2504730830	-1.9317926481	6.9711231104
H	2.8891912936	5.0488410902	2.0776013812
H	-2.9546652615	5.0520111482	-4.4453260526
H	-6.8950611115	1.9573002189	-0.4079777109
H	-6.1781871239	1.9567266320	-2.6774575875
H	-3.0851761039	-3.0750013580	5.4004318088
H	-4.8856594205	-1.5534437255	5.0779006530
H	3.9015265915	3.3834234507	5.1201352677
H	4.1801146605	1.0226093507	5.3116647741
H	1.3022243533	6.2609355322	-2.7832235299
H	1.7278151306	7.0146696742	-0.5620914299
H	-6.9431511311	-1.8982149961	-0.6415467730
H	-5.9946246761	-1.8668044024	-2.8305640124
H	-6.0076561482	-3.4240960396	1.9446632936
H	-4.2523785059	-5.0110984204	2.2369987743
H	-1.1261220549	-4.2683107540	5.3193262742
H	1.1095911072	-5.0671351093	5.1264859133
H	4.3286681026	-1.3456454741	5.1233086827
H	3.7317896234	-3.6251365369	4.8298737253
H	6.0826344429	3.4463483278	1.9415354394
H	6.4345468528	1.0977060015	2.1602235847
H	4.6428605522	4.3511312951	-2.8268045320
H	5.1054058241	5.1007843207	-0.6119526705
H	-0.2471736554	3.5114448810	-6.3440957393
H	-4.7330203240	1.8914639066	-4.5237388865
H	0.6437048613	5.0321223019	-4.7344943837
H	-3.1272290280	1.9148404136	-6.2833083349

FOMO-CASCI/LANL2DZ
Energy = -307.29971813

###

First Excited State Minimum: Geometry and Energies

###

Si10H15

Si	-0.7462658620	-0.2863055414	-2.1799416016
Si	1.5960954923	-0.2528080726	-2.2129231166
Si	-1.5619551257	1.7558412248	-1.3539668488
Si	-1.4636505375	-2.0670640867	-0.8215966591
Si	2.3515664575	0.0915763125	-0.0184084709
Si	-0.7425303080	2.1206532166	0.8171752514

Si	-1.3690489310	-1.9267219084	1.5986218872
Si	1.5995723645	2.1633798656	0.7860420151
Si	-1.4505719556	0.4063037736	2.2636406944
Si	1.4951892960	-1.6069524404	1.3472411127
H	-1.2291811030	-0.4800448492	-3.5637911815
H	2.1125277235	0.8457749476	-3.0503576593
H	2.1295633516	-1.5317062184	-2.7167482855
H	-1.1512124718	2.8604004309	-2.2419216891
H	-3.0365770314	1.7091464710	-1.3143683960
H	-0.7410662471	-3.2836371395	-1.2436092020
H	-2.9029765014	-2.2804167724	-1.0867081512
H	-1.2275509480	3.4280537658	1.3080751225
H	3.8219329377	-0.0240473920	0.0722376039
H	1.7065574526	-2.9897165419	0.8972468049
H	1.7058922360	-1.4670589105	2.7945918754
H	2.1364182576	2.3854672332	2.1412852986
H	2.1135714690	3.2143679820	-0.1116876053
H	-2.8866737289	0.6208855575	2.5441197471
H	-0.7144944741	0.5495913439	3.5355360303

FOMO-CASCI/LANL2DZ
Energy (D0) = -46.11045487
Energy (D1) = -46.09086280

Si22H27

Si	-0.6035630252	0.3476641858	-2.6476810645
Si	1.7435530878	0.3403633331	-2.7302535450
Si	-1.4993386965	2.3194633832	-1.7390597367
Si	-1.2872009879	-1.4445152038	-1.3027111315
Si	2.5163262524	0.6315367466	-0.5331211770
Si	-0.6211891265	2.5415696329	0.4261256136
Si	-0.4710632696	-1.4931887825	1.4256723596
Si	1.7262407105	2.6528413399	0.3708562989
Si	-1.3321821428	0.7552099409	1.7999531892
Si	1.8689664810	-1.1898242784	0.8258452056
Si	2.5107620917	-1.6920619056	-3.6278137445
Si	-3.8455841521	2.1930764382	-1.6801985591
Si	-0.4164569881	-3.5377784826	-1.9777371324
Si	-3.6300163309	-1.5764668770	-1.0086195958
Si	2.8099904350	-3.1425122373	-0.0684039600
Si	2.7685844700	-0.8029571647	2.9837622454
Si	2.5798401447	2.9815095100	2.5343079647
Si	-3.6739079864	0.8046127802	1.9111488465

Si	-0.4369697751	1.1304408703	3.9627325864
Si	-4.5099803187	0.4564355609	-0.2474627083
Si	1.9107361113	1.1877232409	3.8903143325
Si	1.9136661830	-3.4628381819	-2.2071862717
H	-1.1708744382	0.0875857385	-3.9901299050
H	2.1902970429	1.4687427324	-3.5734538353
H	-1.0726373513	3.4616544731	-2.5740061980
H	-1.1494899743	3.7924416423	1.0169451206
H	3.9965372341	0.6623051253	-0.5615583220
H	2.1337892263	3.7665504907	-0.5124219043
H	-4.1162597942	2.1184368886	2.4167429887
H	-4.1709789180	-0.2489096281	2.8148545925
H	-3.9284788102	-2.6744909432	-0.0733749174
H	-4.1680648836	-1.9291601554	-2.3384688054
H	-4.3670673156	1.9496951456	-3.0388273729
H	-4.3910726396	3.4718582137	-1.1867376580
H	-5.9841543941	0.3512060904	-0.2433195686
H	4.0525401228	3.0550939183	2.4809701282
H	2.0610789521	4.2504588683	3.0806075357
H	-0.8992754158	0.0654858724	4.8713500088
H	-0.9417902116	2.4270533059	4.4573784015
H	2.4402290109	1.3757756410	5.2570480497
H	4.2360196223	-0.6893673871	2.8633881477
H	2.4534191754	-1.9474261496	3.8580553722
H	2.4954678274	-4.3042486549	0.7832842912
H	4.2754540542	-2.9930951351	-0.1539597977
H	-1.0727962978	-3.8321751668	-3.2680981522
H	-0.8275734663	-4.5569176187	-0.9971271959
H	1.9187195414	-1.9017042139	-4.9630162970
H	2.3618700471	-4.7434642401	-2.7931922527
H	3.9788444965	-1.6439627002	-3.7642673987

FOMO-CASCI/LANL2DZ
Energy (D0) = -98.20987332
Energy (D1) = -98.18768309

$Si_{26}H_{31}$

Si	-0.8682327074	-0.3519140976	-2.0833286838
Si	1.4836186818	-0.2905091048	-2.2320170815
Si	-1.6904076801	1.7171047127	-1.3144083486
Si	-1.5874752005	-2.1315244811	-0.7051862696
Si	2.2954898311	0.0430382388	-0.0444063416
Si	-0.7736338485	2.0399781779	0.8289018460

Si	-0.7047313040	-2.3582917592	1.5518086779
Si	1.5774472155	2.1262606015	0.7852426022
Si	-1.4534141573	0.2941713153	2.2288054117
Si	1.6422683790	-1.7228208905	1.3838920053
Si	2.1595810125	1.4786518107	-3.6297052190
Si	2.2890612804	-2.3338374776	-3.0803680951
Si	-0.9760482482	3.4686768888	-2.7161092604
Si	-4.0387541298	1.6482873879	-1.1396060695
Si	-0.7393487734	-4.1355081393	-1.6430861546
Si	-3.9312654178	-2.1839582478	-0.7547271198
Si	2.4962814906	-3.7287642904	0.4534322977
Si	2.6282776182	-1.3646831231	3.4810689804
Si	2.3835599489	2.4467807885	2.9753207850
Si	2.2456988642	3.8712957310	-0.6465671784
Si	-3.7982597182	-0.0019373378	2.3150916409
Si	-0.5489567208	0.4298626498	4.4089375528
Si	-4.7147620546	-0.1809851986	0.1676654338
Si	1.7822072952	0.6381223185	4.3462578730
Si	1.6077234278	-4.0936270766	-1.6889599409
Si	1.3694258073	3.5232292883	-2.7961019691
H	-1.4172778060	-0.5823325000	-3.4400586411
H	-1.3123573101	3.2843275317	1.4249468384
H	3.7763364097	0.0568859229	-0.0893331954
H	-4.3122730693	1.1875454513	3.0241718626
H	-4.0925147940	-1.1961344067	3.1256109057
H	-4.4398517333	-3.3267518958	0.0258863285
H	-4.3956830793	-2.3119633736	-2.1495381283
H	-4.6234197848	1.5314103374	-2.4893703346
H	-4.5162691453	2.9048739292	-0.5301353097
H	-6.1877535331	-0.1847494572	0.2925937286
H	3.8537764918	2.5664318281	2.9358152528
H	1.8269153770	3.7000937967	3.5210177106
H	-0.9610271135	-0.7635790594	5.1677654170
H	-1.1814970637	1.6177179850	5.0181361160
H	2.2649738910	0.8762721322	5.7229963556
H	4.0928376543	-1.2696247247	3.3281218016
H	2.3137831428	-2.4771409861	4.3962044099
H	2.1773356519	-4.8544183506	1.3503310271
H	3.9642989735	-3.6050611464	0.3537343512
H	-1.2480289887	-4.2609057335	-3.0236282469
H	-1.2242077189	-5.2836489634	-0.8554893471
H	1.7643646991	-2.5273743817	-4.4463315405
H	2.1174614691	-5.3738525666	-2.2237861624
H	3.7624201372	-2.2763081309	-3.1493899288

H	1.6264505828	1.2524123467	-4.9873545449
H	3.6334762746	1.5036099636	-3.7046429167
H	3.7197095845	3.9137092006	-0.7050065155
H	1.7619117339	5.1541287850	-0.0995979127
H	-1.4986179328	4.7471775955	-2.1953317413
H	-1.5202287077	3.2582090849	-4.0715659062
H	1.8021302741	4.6244949128	-3.6829653076

FOMO-CASCI/LANL2DZ
Energy (D0) = -115.57974181
Energy (D1) = -115.55832364

Si47H49

Si	-1.1311784842	-1.0972652238	-1.5804976960
Si	1.2320179725	-1.0101840103	-1.7037164847
Si	-1.9764714265	0.9534315480	-0.7236902785
Si	-1.7903217458	-2.8957780598	-0.1723114039
Si	2.0921076486	-0.5998789177	0.4672355971
Si	-1.0904062815	1.3053415041	1.4504982362
Si	-1.3583587475	-2.8034138529	2.2234253890
Si	1.2746865758	1.4331797442	1.3676797580
Si	-1.7391727541	-0.4768882451	2.8788651270
Si	1.4057472022	-2.3277261184	1.8804441148
Si	1.9300557680	0.7711788612	-3.0994132284
Si	2.0263317053	-3.1071885861	-2.4801693156
Si	-1.3057362878	2.7253855918	-2.1491052564
Si	-4.3414977822	0.8343393656	-0.5810490162
Si	-1.0467602737	-4.9303475886	-1.0884870918
Si	-4.1599211503	-2.9614417196	-0.0912682281
Si	1.9784319002	-4.4975405394	1.1324179892
Si	2.1735653433	-2.0423141215	4.0891680092
Si	2.1249215909	1.7165524169	3.5671468641
Si	1.9368402841	3.2100577401	-0.0481310769
Si	-4.1171836101	-0.5566103164	2.9588724002
Si	-0.9510115883	-0.0964542787	5.0551711748
Si	-4.9684266239	-0.9585411018	0.8037495932
Si	1.3771430438	-0.0202585694	4.9652397803
Si	1.2952791536	-4.8424112881	-1.0698686195
Si	1.0532407839	2.7665845420	-2.1911771990
Si	-1.8840448685	-1.4475472740	-3.8007208519
Si	-1.9862709547	3.3578334698	2.2246945998
Si	4.4570828112	-0.4769886404	0.4647175390
Si	-4.9337513140	1.4701900122	3.8322354675

Si	-5.3331585970	2.7643166160	0.3237459902
Si	4.4721005748	1.7016749134	3.6905337258
Si	4.5309217707	-2.0642333173	3.9738738458
Si	-1.8975514966	-5.2485878323	-3.2540641825
Si	1.2300819983	-3.6502609815	-4.6262836135
Si	1.0456322607	0.6587493862	-5.2815044057
Si	4.2851004000	0.8216601790	-3.1477676251
Si	4.2703762941	3.3411060546	-0.3511659031
Si	1.0860759823	5.2242897809	0.8277853612
Si	-1.9291797487	4.8628567344	-1.3750158207
Si	-2.1693374546	2.3539739626	-4.3084689405
Si	-4.3325560160	3.2263350276	2.3955972661
Si	-1.1141231396	-3.5154008181	-4.6276556186
Si	5.2657135866	-0.2549617374	2.6669855846
Si	-1.2799537261	0.3651680207	-5.1766560983
Si	-1.2585523230	5.1324012008	0.8569362738
Si	5.1209134270	1.2494457447	-0.9951971401
H	-4.4932027807	-1.6685694576	3.8551898332
H	-4.5775581299	-4.1158370349	0.7244060850
H	-4.6904695384	-3.1313262034	-1.4580940891
H	-4.8710108028	0.5958803576	-1.9403469202
H	-6.4451902637	-0.9881473163	0.8774128019
H	1.6098670907	3.0000966981	4.0878132540
H	-1.4000494249	-1.1843515108	5.9425470699
H	-1.4698884093	1.1842896977	5.5694889444
H	1.9722584680	0.1732145286	6.3052079259
H	1.6981222143	-3.1859530701	4.8927699472
H	1.3822012500	-5.4853614954	2.0474413924
H	3.4489859561	-4.5757007173	1.2490156156
H	-1.4875642516	-6.0285954112	-0.2033898616
H	1.8724074110	-6.1131591261	-1.5589756667
H	3.5040405807	-3.0808479028	-2.4846740715
H	1.4665415342	3.8765178187	-3.0808017056
H	5.0692227047	-1.9343874625	5.3417663484
H	4.9909265867	-3.3424698207	3.4030564357
H	6.7427007697	-0.2431167445	2.6301257359
H	5.0673288678	2.9043118877	3.0887711748
H	4.8444550854	1.6635452974	5.1185582210
H	6.5982742203	1.3004900863	-1.0290183514
H	4.5112943717	4.2919289963	-1.4552596759
H	4.9432878177	3.8599411606	0.8502516679
H	1.5269763351	6.3495014058	-0.0197277048
H	1.6264264228	5.4236508203	2.1859014495
H	-1.8015781321	6.3999438185	1.3914641271

H	-3.3699321548	5.1040381617	-1.5518459474
H	-1.1985976114	5.8462077864	-2.2001884902
H	4.7306317261	1.8899518486	-4.0637543281
H	4.7876092502	-0.4691493863	-3.6548860851
H	1.3278185906	1.9558506879	-5.9289606774
H	1.6990876026	-0.3959354349	-6.0730342523
H	-1.8092987316	0.1270413401	-6.5368968582
H	-3.6411149475	2.2850267446	-4.2393319508
H	-1.7947682756	3.4858237760	-5.1791599058
H	-3.3618083757	-1.5102927615	-3.7844256938
H	-1.6170385285	-3.6930520778	-6.0063014351
H	-1.4316710998	-6.5456677497	-3.7823287327
H	-3.3711042144	-5.2558732247	-3.2174779673
H	4.9800351682	-1.7515121645	-0.0737890793
H	1.8310078814	-2.7960641807	-5.6618839495
H	1.6174989230	-5.0488292584	-4.8987858623
H	-4.8429878496	4.5081712155	2.9255022738
H	-6.4053213704	1.4016821915	3.9255045214
H	-4.3859547819	1.6861217571	5.1833947535
H	-6.7604198246	2.4639386864	0.5543811042
H	-5.2468046463	3.9106092453	-0.5935798254
H	-1.4581114115	3.6060880294	3.5841352096

FOMO-CASCI/LANL2DZ
Energy (D0) = -205.02857344
Energy (D1) = -205.00745851

Si72H63

Si	0.0125318672	-0.0067054838	2.3763559184
Si	0.0440700390	-0.0309445152	4.7487949063
Si	2.2340398421	0.0452120477	1.5632634059
Si	2.2328543183	0.0508619503	-0.8110255308
Si	-1.0773544699	-1.9671859030	1.6163213783
Si	-1.1100460304	1.9330526803	1.5874636677
Si	-1.1116083260	-1.9257759457	-0.7628202941
Si	-1.1219913022	1.9418205460	-0.7945814033
Si	0.1115687181	-3.8827094511	2.3785334365
Si	3.3169502254	-1.8929667405	2.2937282425
Si	0.1535706961	-3.9698590423	4.7262539669
Si	3.3127372544	-2.2061816471	4.6347817484
Si	1.1445589294	-1.9885773041	5.4972796318
Si	2.3554107693	-4.4135211200	1.5269956499
Si	-2.2618450570	0.0008510324	-1.5724644941

Si	1.1143136882	1.9919075929	-1.6065974925
Si	1.1278208653	-1.9103159227	-1.5589513179
Si	-2.2530768719	3.8695561635	-1.5830560013
Si	-2.2586149927	3.8421474854	-3.9504924424
Si	-3.2723494426	1.9159541272	-4.8401004787
Si	-0.0971863286	3.7810645934	-4.8741350320
Si	-2.2526875687	-0.0111040820	-3.9461641840
Si	1.0984033130	1.9581858149	-3.9792586601
Si	-0.0132828008	-0.0004125013	-4.6708260665
Si	2.3008639137	-3.8369676765	-0.8307528290
Si	1.1172712020	-1.9255506884	-3.9277242823
Si	4.4827675515	-3.8110051358	-1.7273814007
Si	1.1386725091	-5.7475839333	-1.6521625363
Si	0.0009142052	-3.8617503652	-4.6973315917
Si	3.3359901086	-1.9041710203	-4.7385194891
Si	1.1578938592	-5.7770051104	-3.9991175867
Si	4.4489204126	-3.8556311862	-4.0700381231
Si	-2.1881742326	0.0098614883	5.5354326429
Si	1.1354260006	1.9226421230	5.5149405878
Si	-3.3230692539	1.9501583909	2.4131377906
Si	-0.0145611079	3.8678459825	2.3936268470
Si	-4.4605672600	3.8592904753	-0.7274191655
Si	-1.1445635542	5.7890104358	-0.7484920924
Si	-1.1647138379	5.7782135401	1.6068664999
Si	-0.0123883387	3.8352812366	4.7574816695
Si	-3.2707987139	1.9573486125	4.7771523823
Si	-4.4208551963	3.8901394478	1.6270317457
Si	-2.2164193148	3.9016788051	5.5605885152
Si	-3.3510607704	5.8069089250	2.4588521782
Si	-3.3328706783	5.8207001083	4.8043668346
Si	-3.2053008073	-1.9520076940	-4.8804727261
Si	-4.4803963138	0.0106918535	-0.7332736593
Si	-2.2448758713	-3.8543108526	-1.5455875232
Si	-2.2187224248	-3.8503157471	-3.9129280850
Si	-0.8774556899	-5.8764072457	1.5351449176
Si	-1.0603558366	-5.7646038883	-0.8025116011
Si	-3.3134445569	-1.9441255893	2.4260875320
Si	-4.3784156340	0.0022070501	1.6188578579
Si	-5.6634206330	-1.9234104941	-1.3765593997
Si	-4.5964086866	-3.7521885781	1.6250605515
Si	-4.4651759062	-3.8385436939	-0.7221959351
Si	3.3491482382	-5.7801545034	-4.8352429920
Si	3.3648681866	1.9648234911	2.3808757020
Si	2.2160461682	3.9246738031	-0.7766996405

Si	2.1914363858	3.8460613849	1.5779915131
Si	5.5571849547	-1.9020234882	-0.8787109521
Si	4.4266852417	0.0370802957	-3.9684866927
Si	4.4661411413	0.0708864553	-1.5995967137
Si	5.5704011134	2.0973882182	1.5625644260
Si	4.4822342825	3.9983256497	-1.4180931236
Si	5.5584041765	2.0050336820	-0.7862009164
Si	3.3388325721	2.1282090290	4.7303877683
Si	-3.5035855228	-1.7786359045	4.7686015432
Si	-5.6947699575	1.9458887565	-1.3055884116
Si	1.1209008379	5.9280975623	-1.3546282183
Si	5.4944444113	-2.2471196724	1.4376061376
Si	3.2532065244	1.8353048781	-4.9208779426
H	3.3197787098	-5.7905163266	-6.3102415905
H	4.0666255759	-6.9812077075	-4.3671167232
H	0.4322733273	-6.9773405331	-4.4666063304
H	5.8391979635	-3.8204685191	-4.5716084679
H	5.1978589079	-4.9966394246	-1.2091035805
H	3.2769256639	-1.8366380490	-6.2153629517
H	1.8393563308	-6.9419448379	-1.1342264693
H	-0.0313790686	-3.8041479729	-6.1752952050
H	5.8251330674	0.0318258280	-4.4510126595
H	6.9668795335	-1.8616660454	-1.3227606288
H	-1.7535474314	-6.9707904735	-1.3042200984
H	-2.9016529929	-5.0742992985	-4.3861841430
H	-2.1790084182	-6.1022867005	2.1831086245
H	0.0110587824	-6.9988048106	1.8862371377
H	5.8706058743	-3.6432510650	1.7215420595
H	6.3974896537	-1.3674638087	2.2005552350
H	6.9492573733	2.0069429684	-1.2890014255
H	3.9784048142	3.1092220981	-4.8011509874
H	3.0809921180	1.5431992918	-6.3581692351
H	-0.0214956162	-0.0047409291	-6.1511507439
H	-4.6718392418	-1.9529959812	-4.7725367490
H	-2.8557713782	-1.9512348519	-6.3154084995
H	-5.1538551694	-5.0503188846	-1.2174031149
H	-4.7158787325	5.8275023436	5.3179052889
H	-2.6404856573	7.0288880693	5.2918662861
H	-4.0477846123	7.0061134387	1.9469314846
H	-2.1902297388	3.8716856893	7.0384052628
H	0.7282381390	5.0294383066	5.2208588828
H	-0.4285323347	6.9713990678	2.0797029112
H	-5.8174096782	3.8482414381	2.1133838727
H	-4.6702319311	1.9223351505	5.2558418269

H	-5.1529144380	5.0731031876	-1.2126295191
H	-1.8671025520	6.9883015306	-1.2257862939
H	1.1301556065	1.9117041112	6.9941350121
H	-2.1524138751	0.0207857474	7.0143086163
H	-5.7690464509	-0.0173136828	2.1256259145
H	1.2098201216	-1.9641532308	6.9740894999
H	2.8924997484	5.0521273999	2.0728742801
H	-2.9712492538	5.0471455494	-4.4277435330
H	-6.9202896672	1.9415934580	-0.4814111068
H	-6.1003338232	1.9423033487	-2.7192019664
H	-3.1948502581	-3.0428159346	5.4525527328
H	-4.9128063670	-1.4418056463	5.0549555764
H	3.8272802586	3.4777621486	5.0766171650
H	4.2435306322	1.1456515501	5.3484534892
H	1.3081345264	6.2627997903	-2.7743988084
H	1.7340314221	7.0033939355	-0.5489892801
H	-6.9429321763	-1.9234248868	-0.6391117423
H	-5.9703939076	-1.9043954950	-2.8142631954
H	-6.0037965274	-3.4699720872	1.9736291676
H	-4.2186685634	-5.0175138961	2.2708120406
H	-1.1918455618	-4.1934134679	5.2727804598
H	1.0148469566	-5.0945818316	5.1366517407
H	4.2818915203	-1.2522943705	5.1997003085
H	3.8015086575	-3.5700937824	4.9065355178
H	6.1191974441	3.4065894824	1.9685351691
H	6.4122535273	1.0477350983	2.1585114143
H	4.6262191552	4.2875559347	-2.8524114660
H	5.1139262816	5.1015081142	-0.6659290849
H	-0.2391996710	3.5436268660	-6.3249250044
H	-4.7283871422	1.8938322732	-4.6370271126
H	0.6362832366	5.0425339800	-4.6921029305
H	-3.0168560700	1.9114066136	-6.2947984620

FOMO-CASCI/LANL2DZ
Energy (D0) = -307.31982928
Energy (D1) = -307.30140058
